# Highly effective sequestration of Ni(II) and Cr(III) ions from aqueous solution using foamed and non-foamed metakaolin based geopolymer

**DOI:** 10.1038/s41598-025-24676-3

**Published:** 2025-11-10

**Authors:** Fatma A. Mohammed, Sara H. Abdullah, Essam A. Kishar, Doaa A. Ahmed, Nourhan N. Kassem

**Affiliations:** Chemistry Department, Faculty of Women for Arts, Science and Education,, Ain Shams University, Cairo, 11757 Egypt

**Keywords:** Heavy metal, Adsorption, Porous geopolymer, Equilibrium isotherms, Sustainability, Chemistry, Engineering, Ecology

## Abstract

Heavy metals are considered one of the most hazardous materials, which can cause serious threats to the environment and living organisms. Their toxicity, persistence, and bio accumulative nature make them a prime environmental concern. The present study aims to develop novel adsorbents fabricated from 80% metakaolin and 20% fly ash for producing foamed and non-foamed geopolymer composites, tailored to remove Ni(II) and Cr(III) from an aqueous solution. The synergistic effects emanating from this unique amalgamation of materials present a promising avenue for the effective remediation of Ni(II) and Cr(III) contamination in aqueous environments. The synthesized adsorbents before and after adsorption were characterized by SEM, FTIR, BET, and XRD to provide a better insight into the adsorption process. The experimental data indicated that the maximum adsorption capacities of the non-foamed geopolymer for Ni(II) and Cr(III) were determined to be 28.34 mg/g and 29.81 mg/g, respectively. While, in the case of the foamed geopolymer, the maximum adsorption capacities of 38.46 mg/g and 39.42 mg/g were recorded for Ni(II) and Cr(III), respectively, at an initial concentration of 200 mg/L. Compared to other commonly used adsorbents reported in the literature, such as activated carbon (17.24 mg/g), natural zeolites (7.90 mg/g), and metakaolin-based geopolymer (10.2 mg/g), it was observed that the foamed geopolymer developed in this study exhibits markedly superior adsorption performance for both Ni(II) and Cr(III) ions. According to the obtained results, the porous geopolymer achieved a maximum removal efficiency of 91.67% and 97.21% at 25 °C, pH 5.0, 120- and 180-min contact time, 0.1 g/L adsorbent dosage, and a preliminary concentration of 5 mg/L for Ni(II) and Cr(III), respectively. The adsorption kinetics followed the pseudo-second-order kinetics, whereas the adsorption data fitted well with the Freundlich isotherm model, which indicated that the sorption mechanism is mainly chemisorption. The negative values of ΔG° indicated the spontaneous nature of the adsorption process, and the positive values of enthalpy (ΔH°) values confirm that the adsorption is endothermic. The FTIR analysis confirmed shifts and intensity changes in functional groups such as OH and Si–O–T after adsorption, indicating chemical interaction with heavy metals. Additionally, SEM images revealed morphological differences in both sorbents, suggesting the entrapment of Ni(II) and Cr(III) onto the sorbent after adsorption. The findings suggest that the prepared geopolymer proves to be a promising cost-effective adsorbent for the remediation of heavy metal contamination in industrial wastewater.

**Supplementary Information:**

The online version contains supplementary material available at 10.1038/s41598-025-24676-3.

## Introduction

In the present scenario, the rapid growth of industrialization has led to significant dramatic environmental degradation, including water pollution. For instance, heavy metals are particularly regarded as one of the most hazardous materials owing to their toxicity and ability to bioaccumulate in living cells, posing severe health risks and may be lethal to humans^1^. The presence of heavy metals in water, even at lower levels, can cause critical health issues such as cancer, nephrotoxicity, and organ damage for all living organisms^2,3^. Indeed, the discharge of industrial effluent containing several heavy metals is a significant contributor to exacerbating this environmental problem. Among all of the existing heavy metals, nickel Ni(II) and chromium Cr(III) are prevalent toxic pollutants, especially when exceeding the permissible levels. The main sources of nickel are electroplating industries, rubber and plastic industries, and nickel-cadmium battery production^4^, with excessive intake leading to serious health impacts, such as skin irritation, neurological disorders, gastrointestinal distress, and lung and bone cancer^5,6^. Chromium is the other toxic heavy metal and is extensively used in various industries like paints and pigments, leather tanning, ceramic industries, textile and metal finishing^7^. Exposure to excess chromium is likely to cause skin allergy, lung cancer, stomach problems, and respiratory diseases^8,9^. Thesepollutants are predominantly discharged onto the water by several industrial processes like the refining of ores, nickel alloy production, and electroplating. Hence, it is crucial to remove these heavy metals to clean contaminated water. Numerous methods have been utilized and implemented to remove heavy metals from wastewater, such as flotation, ion exchange, adsorption, electrochemical processes, membrane bioreactors, precipitation, photocatalysis, and filtration. These conventional methods have limitations such as high reagent and energy requirements, high costs, and inefficiency at low concentrations of heavy metals, etc. In contrast, the adsorption method is considered sustainable and more efficient in the removal of heavy metals from aqueous media because of the benefits of simple operation and cost-effectiveness^10^. Therefore, in this study, we explore the development of innovative and eco-friendly adsorbents based on metakaolin and fly ash as aluminosilicate sources to produce foamed and non-foamed geopolymer cement tailored for removing toxic Ni(II) and Cr(III) ions from aqueous environments by using an economical adsorption method. The synthesized adsorbents demonstrated promising capabilities in removing Ni(II) and Cr(III) pollutants, making them a suitable candidate for removing other poisonous metal ions in contaminated water.

Porous geopolymer has emerged as a promising avenue for removing a wide array of heavy metals such as Pb(II), Cd(II), and Ni(II) from wastewater due to their beneficial properties, including high specific surface area, porous structure, and their chemical stability^11,12^. The experiment done by Pang et al.^13^ showed that when the specific surface area of the adsorbent increased, the adsorption ability improved. According to Yuanyuan Ge et al.^14^ the porous metakaolin-based geopolymer sphere demonstrated superior removal effectiveness for Cu(II) ions in comparison to the commercial sphere. Novais et al.^15^ prepared porous geopolymer monoliths by combining metakaolin (MK) and fly ash (FA) as a source of aluminosilicate to sequester Pb)II) in aqueous solution. The common foaming agents used in the geopolymer composite are aluminum (Al) powder^16,17^, silicon (Si) powders^18–20^, and hydrogen peroxide (H_2_O_2_)^21,22^. Several investigations have revealed that hydrogen peroxide is preferred as a foaming agent due to its well-controllable decomposition and more homogeneous distribution within the geopolymer slurry^23–25^. The hydrogen peroxide foaming principle is demonstrated in Eq. (1):

1 $$2{{\text{H}}_2}{{\text{O}}_2} \to {{\;}}2{{\text{H}}_2}{{O\;}} + {{\;}}{{\text{O}}_2} \uparrow$$

Many researchers were restricted to using pure metakaolin in the synthesis of geopolymers as an effective adsorbent. For instance, Kara et al.^26^ studied zinc(II) and Ni(II) ion removal by using bulk metakaolin-based geopolymer and reported that the adsorption capacity values for Ni^+2^ and Zn^+2^ were found to be 42.61 mg/g and 74.53 mg/g, respectively. Karima, et al.^27^ revealed that the use of Algerian Tamazert kaolin as an adsorbent for Cr(III) removal from wastewater was achieved exceptional Cr(III) adsorption ability with 99.11% removal efficiency under ideal circumstances. Haize Jin et al.^28^ synthesized metakaolin geopolymer for the adsorption of Ni(II) ions from aqueous solutions. They reported the maximum adsorption capacity as 8.3 mg/g. T.W. Cheng et al.^29^ successfully adsorbed different heavy metals (Pb^+2^, Cu^+2^, Cr^+3^, and Cd^+2^) by employing a metakaolin-based geopolymeric binder and recorded the maximum adsorption capacity of 10.2 mg/g for Cr^+3^. S. Andrejkovičová et al.^30^ investigated the efficiency and adsorption of Pb(II), Zn(II), Cu(II), Cd(II), and Cr(III) metal solutions in the metakaolin-based geopolymers. They recorded the maximum sorption capacity of 21.8 mg/g Cr^+3^ for MK100 geopolymer. According to Kamel et al.^31^, the simultaneous removal of Cr^+3^ on volcanic tuff-based geopolymer achieved an uptake capacity of 19.3 mg/g of geopolymer. Further studies on the adsorption of Ni(II) using geopolymeric adsorbent derived from LD slag show the maximum adsorption capacity for Ni(II) was reached at 14.72 mg/g by Chayan Sarkar et al.^3^.

Therefore, the distinctive contribution of our work lies in the synergistic effects emanating from the combination of 80% metakaolin (MK) and 20% fly ash (FA) to fabricate foamed and non-foamed MK/FA-based geopolymers, tailored to remove Ni(II) and Cr(III) ions from an aqueous solution. It is worth highlighting that, to the best of our knowledge, no previous investigation has specifically delved into the adsorption of Ni(II) and Cr(III) ions using this unique amalgamation of materials. This underscores the novelty of our research, presenting a promising avenue for addressing Ni(II) and Cr(III) contamination in water through the innovative composition of our foamed and non-foamed metakaolin- fly ash geopolymer composite. This work meticulously appraises the efficacy of these adsorbents via a series of batch adsorptions, such as sorbent dosage, solution pH, contact time, and initial concentration, followed by isotherm and kinetic models, supplemented by various characterization methods. Moreover, the study aims to evaluate the thermodynamic behavior of the adsorption process by determining the thermodynamic parameters such as Gibbs free energy (ΔG°), enthalpy (ΔH°), and entropy (ΔS°), to provide an idea about the nature of adsorption.

## Experimental design

### Materials

The calcined kaolin and fly ash were used as the aluminosilicate source material in the fabrication of porous geopolymer supplied by Nile Oversees Company (Cairo, Egypt). The chemical composition of the raw materials was determined by X-ray fluorescence (XRF) and is given in Table 1. The metakaolin consists predominantly of silica (SiO_2_) (52.23%), alumina (Al_2_O_3_) (27.65%), calcium oxide (CaO) (14.73%) and iron (III) oxide (Fe_2_O_3_) (2.26%), with less than 2.00% for other oxides. The alkaline activator solution was a mixture of 10 M sodium hydroxide (NaOH) and liquid sodium silicate (Na_2_SiO_3_) with a ratio of 1:2.5. Previous studies have shown that this ratio is extremely suitable for achieving good properties of the binder^27,32^. The NaOH pellets were purchased from ELGoumhouria chemical company, Cairo, Egypt have (99%) purity with a density of 2.13 g/cm^3^, while the liquid Na_2_SiO_3_ contains 11.7% Na_2_O, 32.8% SiO_2_ and 55.5% H_2_O. Commercial liquid sodium silicate was purchased from Silica Egypt Company, Burg Al-Arab, Alexandria, Egypt. Hydrogen peroxide (H_2_O_2_) (30% wt./wt.) was utilized as a foaming agent to produce porous geopolymer. Standard stock solutions of Ni(II) and Cr(III) (1000 ppm) were prepared by dissolving a required quantity of NiCl_2_⋅6H_2_O (M.wt. of 237.69108 g/mol and 98% purity) and CrCl_3_.6H_2_O (M.wt. of 266.4468 g/mol and 98% purity) in distilled water. All the chemicals of analytical grade were purchased from (Universal Laboratories,507, Raheja Centre, Mumbai, India). The chemical composition of each raw material was carried out using an X-ray fluorescence spectrometer (S6 JAGUAR, Bruker).

**Table 1:** Chemical compositions of Metakaolin and fly Ash (wt%).

Materials	SiO_2_	Al_2_O_3_	Fe_2_O_3_	MgO	SO_3_	CaO	K_2_O	Na_2_O	Cl^−^	LOI
Metakaolin	52.23	27.65	2.26	0.96	0.13	14.73	0.8	0.1	0.22	0.73
Fly ash	38.16	17.20	14.90	4.76	1.71	18.10	1.84	1.18	0.06	0.24

### Synthesis of a porous geopolymer

The foamed geopolymer paste was prepared following the aforementioned steps using 10 M NaOH and liquid sodium silicate Na_2_SiO_3_ at a ratio of 1:2.5. First, calcined kaolin (MK) and fly ash (FA), selected as the precursor materials, were mixed in a weight ratio of 4:1 and then combined with the alkaline activator. An alkaline activator (prepared one day before to allow heat release) and water were added. Then, the foaming agent H_2_O_2_ (1.2% by weight) was added to the paste. Likewise, a control non-foamed geopolymer sample was synthesized using the same method of preparation used for foamed geopolymer, except that H_2_O_2_ was not added. After the completion of mixing, the slurry was immediately poured into cubic molds with a dimension of (25 × 25 × 25 mm) and then left undisturbed in humidity (100% R.H.) under room temperature for 24 ± 1 h to obtain the foamed MK/FA geopolymer (FMFG) and similarly, the un-foamed geopolymer pastes were denoted (UFMFG). Upon de-molding, the samples were kept and cured at ambient temperature until 28 days to ascertain the influence of foaming on the Ni(II) and Cr(III) removal. Therefore, before the batch adsorption test, the geopolymer samples were crushed to ≤ 150 μm, washed with distilled water to eliminate excessive alkalis until the washing water had a neutral pH, and dried in an oven at 80 °C for 24 h. Excessive pH may result in the precipitation of metal ions due to the formation of hydroxides such as Ni (OH)_2_ and Cr (OH)_3_, which will influence the adsorption process.

### Characterization

The compressive strength of MFG pastes was obtained by a D550-control type (Milano-Italy machine). The surface morphology and internal microstructure were observed under a scanning electron microscope (SEM) (ESEM/Mapping: FEI, model Quanta FEG 250) was used, at 15 kV to investigate the microstructure of the geopolymers before and after adsorption. Fourier-transformed infrared spectroscopy (FTIR) (PerkinElmer 1430 infrared spectrophotometer, USA) was used to identify the functional groups of feedstocks geopolymer composites before and after Ni(II) and Cr(III) adsorption, which operate in the wavenumber range 400–4000 cm^−1^ using the KBr disk method. The mineralogical composition of the raw materials and the synthesized geopolymers was characterized using X-ray diffraction (XRD) (Bruker D2 phaser 2nd generation X-ray diffraction) Cu Kα, radiation (the wavelength was 1.54184 Å) with a 30 kV generator and a 10 mA. Analysis of each powder sample (ɸ ≤ 75 μm) was performed over the 2θ range from 5^o^ to 70^o^ and a scan rate of 2 degrees/min. The point of zero charge (pH_pzc_) was obtained using the protocol described by Karadag^33^. Brunauer-Emmett-Teller (BET) specific surface area and pore size distribution were measured by using an automatic surface area and pore size analyzer calculated with N_2_-gas adsorption at 77 K (BELSORP MINI X). The concentration of heavy metal ions before and after the adsorption process was analyzed by an atomic absorption spectrometer (GBC model Savant AA). A digital pH/mV meter (Model AD1000, Adwa Instruments, Hungary) was used to monitor and adjust the pH of metal ion solutions during adsorption experiments. The instrument was calibrated using standard buffer solutions (pH 4.00, 7.00, and 9.00) before each batch of measurements to ensure accuracy. An orbital shaker with temperature control (Köttermann, Germany) was used to maintain uniform agitation of the adsorption mixtures at 200 rpm and 25 ± 1 °C throughout the experimental period.

### Batch adsorption study

Batch adsorption techniques were carried out to ascertain the effectiveness of the foamed geopolymer in removing Ni(II) and Cr(III) ions from aqueous solution and compared the results with the non-foamed geopolymer. The experiment was carried out at room temperature, and variable parameters were investigated. In each experimental run, 100 mL of synthetic either Ni(II) or Cr(III) solution was taken in a 150 ml conical flask containing 0.1 g of each adsorbent and shaken at 200 rpm for time intervals of 5, 10, 15, 30, 60, 120, 180, and 240 min. The solution pH (1–9), adsorbent dosage (0.02–0.2 g), and initial metal ion concentrations (5, 10, 30, 50, 100, and 200 mg L^−1^) were varied to study their effects on the metal ion removal efficiency. Different concentrations were prepared by diluting the Ni(II) or Cr(III) stock solution, while the pH of the solution was adjusted using 0.01 M NaOH or 0.01 M HCl solution. The initial Ni(II) and Cr(III) concentration was adopted based on the designed parameter, while the final concentration was detected by using atomic absorption spectrophotometry (AAS). The metal uptake and the percentage removal were calculated by using Eqs. (2) and (3)^34^.

2 $${q_e} = \frac{{{c_o} - {c_e}}}{m} \times v$$

3 $${{RE\;}}\left( {{\% }} \right) = \left( {\frac{{{{\text{C}}_{\text{o}}} - {{\text{C}}_{\text{e}}}}}{{{{\text{C}}_{\text{o}}}}}} \right) \times 100$$

Where:

q_e_: uptake of adsorbate per unit mass of adsorbent (mg/g).

C_o_: starting concentration of Ni(II) or Cr(III) in the aqueous solution (mg/L).

C_e_: the final equilibrium concentration of solution (mg/L).

m: adsorbent mass (g).

V: sample volume(L).

RE (%) represents the Ni(II) or Cr(III) removal efficiency.

### Adsorption isotherm models

To investigate the adsorption isotherms, the experiments were performed by adding 100 mL of varying initial concentrations of Ni(II) or Cr(III) from 5 mg L^−1^ to 200 mg L^−1^ under optimized conditions of pH 5, contact time of 120 min for Ni^+2^ and 180 min for Cr^+3^, and a dosage ofadsorbents of 0.1 g/L in this study. The pH of the mixture solution was adjusted to pH = 5 ± 0.1 by using 0.01 M NaOH or 0.01 M HCl solution. The study examines two models of isotherm namely, the Langmuir and Freundlich isotherm models, which were applied to fit the equilibrium isotherm data. The Langmuir isotherm model describes adsorption as a monolayer process occurring on a uniform surface with identical and energetically equivalent active sites. In contrast, the Freundlich isotherm assumes adsorption takes place on a heterogeneous surface, allowing for multilayer formation and varying affinities across the adsorption sites. The linear equations of Langmuir and Freundlich models that can be express as following Eqs. (4) and (5), respectively^34^.

4 $$\frac{{{c_e}}}{{{q_e}}} = \frac{1}{{{q_m}{k_L}}}\frac{1}{{{q_m}}}{c_e}$$

5 $${{Ln\;}}{{\text{q}}_{{e}}} = {{Ln\;}}{{\text{K}}_{\text{F}}} + \frac{1}{{\text{n}}}{{Ln\;}}{{\text{c}}_e}$$

where: (C_e_) is the equilibrium concentration of selected metals in mg/L, (q_e_) is the uptake capacity at equilibrium in mg/g, (q_m_) is the maximum adsorption capacity in mg/g, (n) denotes the absence of linearity of the adsorbed quantity in the function of C_e_, (K_L_) is Langmuir constant for adsorption in L/mg, and (K_F_) is Freundlich constant in mg/g.

### Kinetics adsorption models

The experiment was done to determine the kinetics of adsorption. The 0.1 g of geoadsorbents was added into 100 mL single solutions of Ni(II) or Cr(III) with an initial metal concentration 5 mg/L (25 ± 1 °C, pH = 5 ± 0.1). The times were intervals of 5, 10, 15, 30, 60, 120, 180, and 240 min. Kinetic models are employed to elucidate the mechanism of the adsorption process. The pseudo-first-order and pseudo-second-order models are commonly applied to assess the adsorption kinetics, providing insight into the nature of the interaction between adsorbate and adsorbent, whether it is primarily physical or chemical in nature. Their equations are given by Eqs. (6) and (7), respectively^34^.

6 $${{Ln\;}}({{\text{q}}_{\text{e}}} - {{\text{q}}_{\text{t}}}) = {{Ln\;}}{{\text{q}}_{\text{e}}} - {{\text{K}}_1}t$$

7 $$\frac{{\text{t}}}{{{{\text{q}}_{\text{t}}}}} = \frac{1}{{{{\text{K}}_{2{{\;}}}}{{\text{q}}_{\text{e}}}^2{{\;}}}} + \frac{1}{{{{\text{q}}_{\text{e}}}}}t$$

where: (q_e_) is the uptake capacity at equilibrium time in mg/g, (q_t_) is the uptake capacity at the given time in mg/g, and (t) is the contact time in min. k_1_ (1/min) and k_2_ (g/mg/min) are the rate constant of the corresponding model.

### Thermodynamic adsorption

The experiment method was carried out at different temperatures 25 °C, 35 °C, and 45 °C. The dosage was 0.1 g, and the pH was 5. The thermodynamic parameters were determined by using the following equations:

8 $$\Delta G^\circ \; = {\text{ }} - RTln{K_d}$$

9 $$K = \frac{{{q_e}}}{{{C_e}}}$$

10 $$\Delta G^\circ = \Delta H^\circ - {\text{ }}T\Delta S^\circ$$

11 $$lnK{\text{ }} = \frac{{\Delta {\text{S}}^\circ }}{{\text{R}}} - \frac{{\Delta {\text{H}}^\circ }}{{{\text{RT}}}}$$

where, q_e_ is the amount of heavy metal adsorbed on the adsorbent of the solution at equilibrium (mol/L), C_e_ is the equilibrium concentration (mg/L), ∆G° is the standard Gibbs free energy, ∆H° and ∆S° are the enthalpy and entropy change of adsorption process. K is the adsorption equilibrium constant, “R” is the universal gas constant (8.314 J·mol^−1^·K^−1^) and T is the solution temperature (K).

## Results and discussion

### Raw materials characterization

**Fig. 1:**
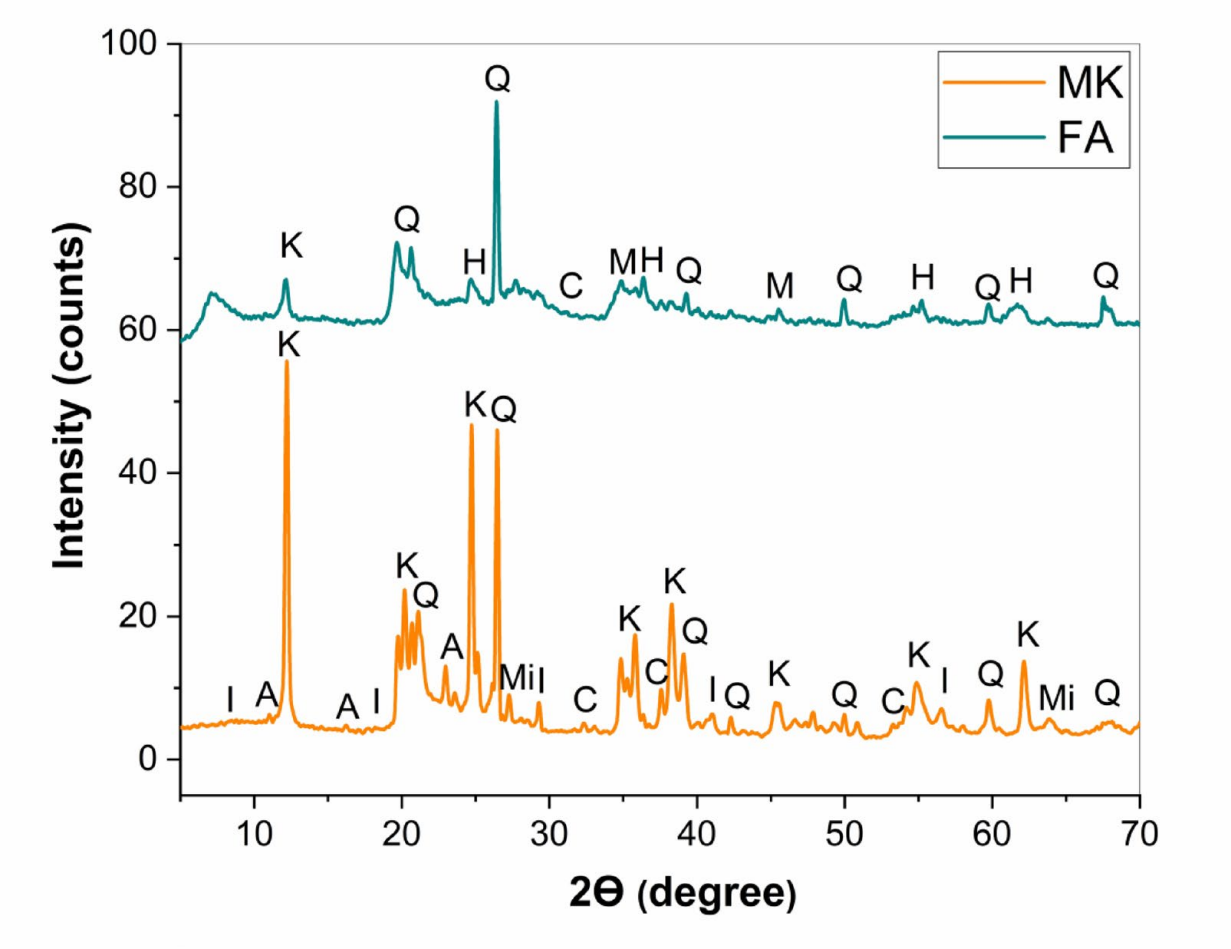
X-ray diffraction patterns of raw materials; (K: Kaolinite, I: Illite, M: Magnetite, Mi: Microcline, A: Aluminium silicate, C: Calcium oxide, H: Hematite, and Q: Quartz).

**Fig. 2:**
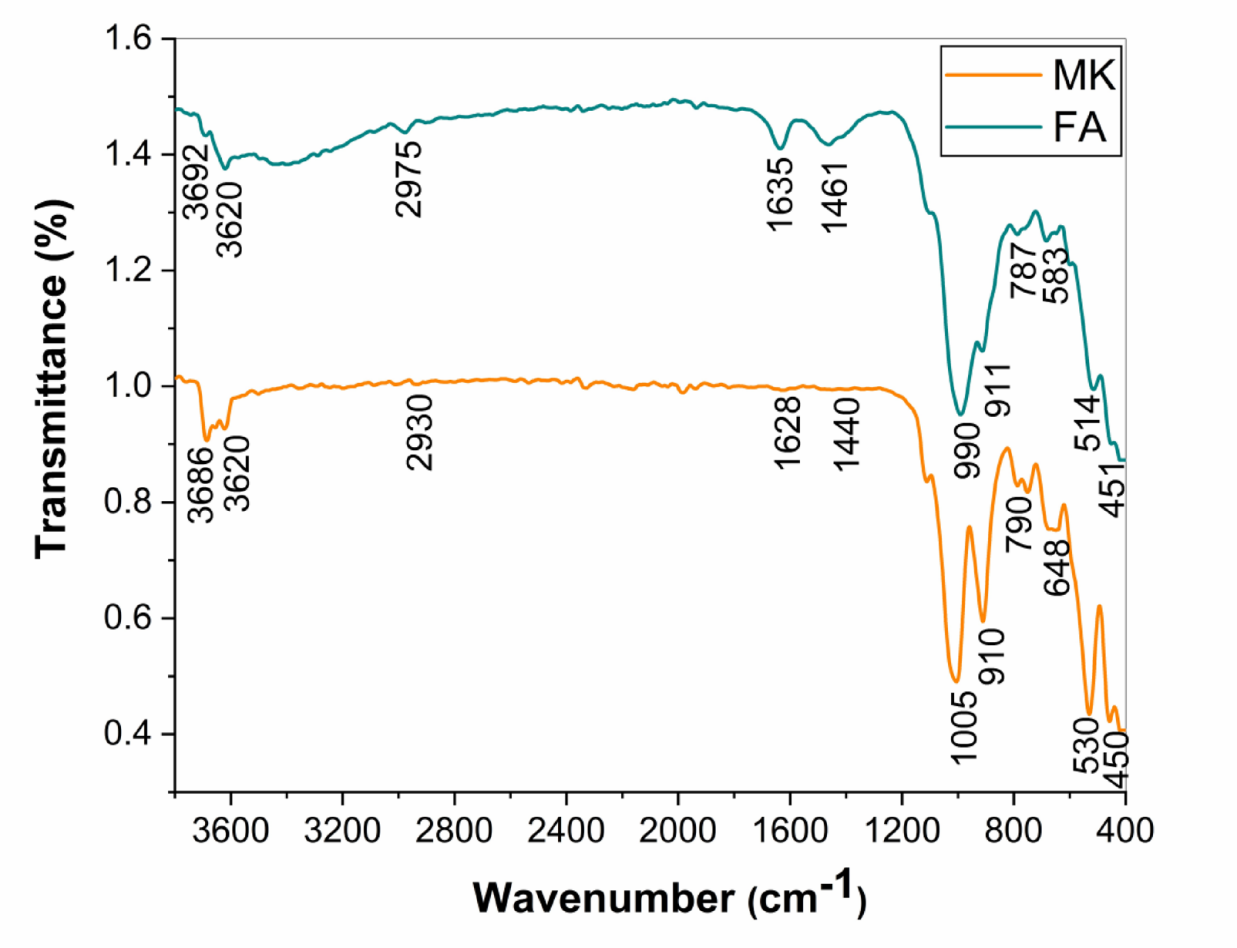
FT-IR spectra of different raw materials.

Figure 1 depicts the X-ray diffraction patterns of MK and FA as raw materials. Metakaolin diagram exhibited main characteristic peaks of kaolinite were observed at 2Ө 12.35, 24.88^o^,19.80 ^o^, 20.38^o^, 34.93^o^, 35.93^o^, 38.35^o^, 45.24^o^, 54.88^o^, and 62.19^o^. The intense and narrow peaks confirm that the MK was well crystallized. The MK also contained other minerals such as quartz at 2Ө 26.63^o^, microcline, calcium oxide, and illite^17,35^. Furthermore, FA demonstrates a prevailing amorphous structure with major peaks of mullite, haematite, and quartz^36^. The FTIR spectra of different raw materials are presented in Fig. 2. It can be noticed that the IR spectrum of MK shows the band appearing at 1628 cm^−1^ is attributed to the bending vibration of O-H water molecules. The bands of both MK and FA are observed at 450 cm^−1^ and 451 cm^−1^, respectively, corresponding to the bending vibration of Si-O-Si. The stretching vibration of H-O-H is detected at 3620 cm^−1^ in both materials. Another intense band associated with asymmetric stretching vibration of Si-O-T is recognized in both MK and FA at 1005 cm^−1^ and 990 cm^−1^, respectively. The last bands that appeared in the ranges of 583–648 cm^−1^ and 514–530 cm^−1^ are attributed to symmetrical stretching vibrations of Si–O–Si, Si–O–Al, and Si–O, respectively^37^.

### Characterization of geopolymer specimens

#### Scanning electron microscope (SEM)

**Fig. 3:**
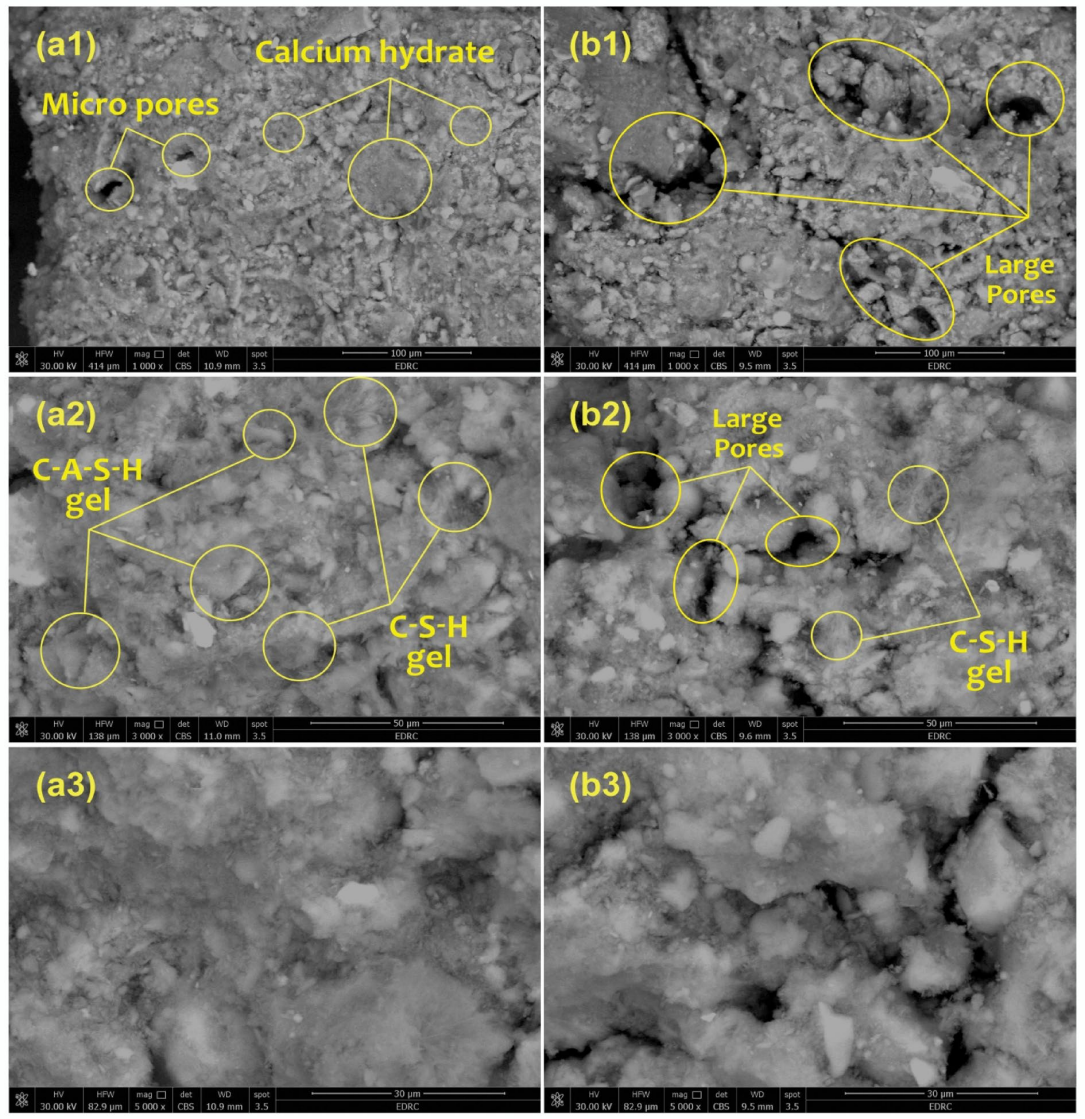
SEM image of (**a**) UMFG: (a1) 1000 x (a2) 3000 x (a3) 5000 x and (**b**) the FMFG: (b1) 1000 x (b2) 3000 x (b3) 5000 x.

The microstructure and surface morphology of the UMFG and FMFG geopolymer specimens are displayed in Fig. 3. After comparing the SEM images of both samples under the same magnification, it was observed that the un-foamed geopolymer surface (UMFG) was shown to have a compact gel and dense structure. Consequently, there might be fewer adsorbable active sites, which could lead to less interaction with adsorbates^34^. While the addition of H_2_O_2_ to the geopolymer increased the generation of endogenous gases and, as demonstrated, created more pores with varying sizes and morphologies. Also, it was found that the pores in the foamed geopolymer specimens were pronounced and wider than those in the non-foamed geopolymer samples. Figure 3 shows discernible distinctions between the un-foamed (UMFG) and foamed (FMFG) geopolymer samples. It is worth noting that the SEM images of the FMFG sample clearly capture the bubbles that were produced during the foaming process (Fig. 3b1**)**. The microstructures are in line with the findings of the FTIR and XRD analyses. The porosity increases as H_2_O_2_ is added to the geopolymeric network, which increases the specific surface area. However, one of the benefits that might support the simultaneous sequestration of Ni(II) and Cr(III) is the increase in specific surface area of the geopolymer structure^38^.

#### The infrared spectra (IR)

**Fig. 4:**
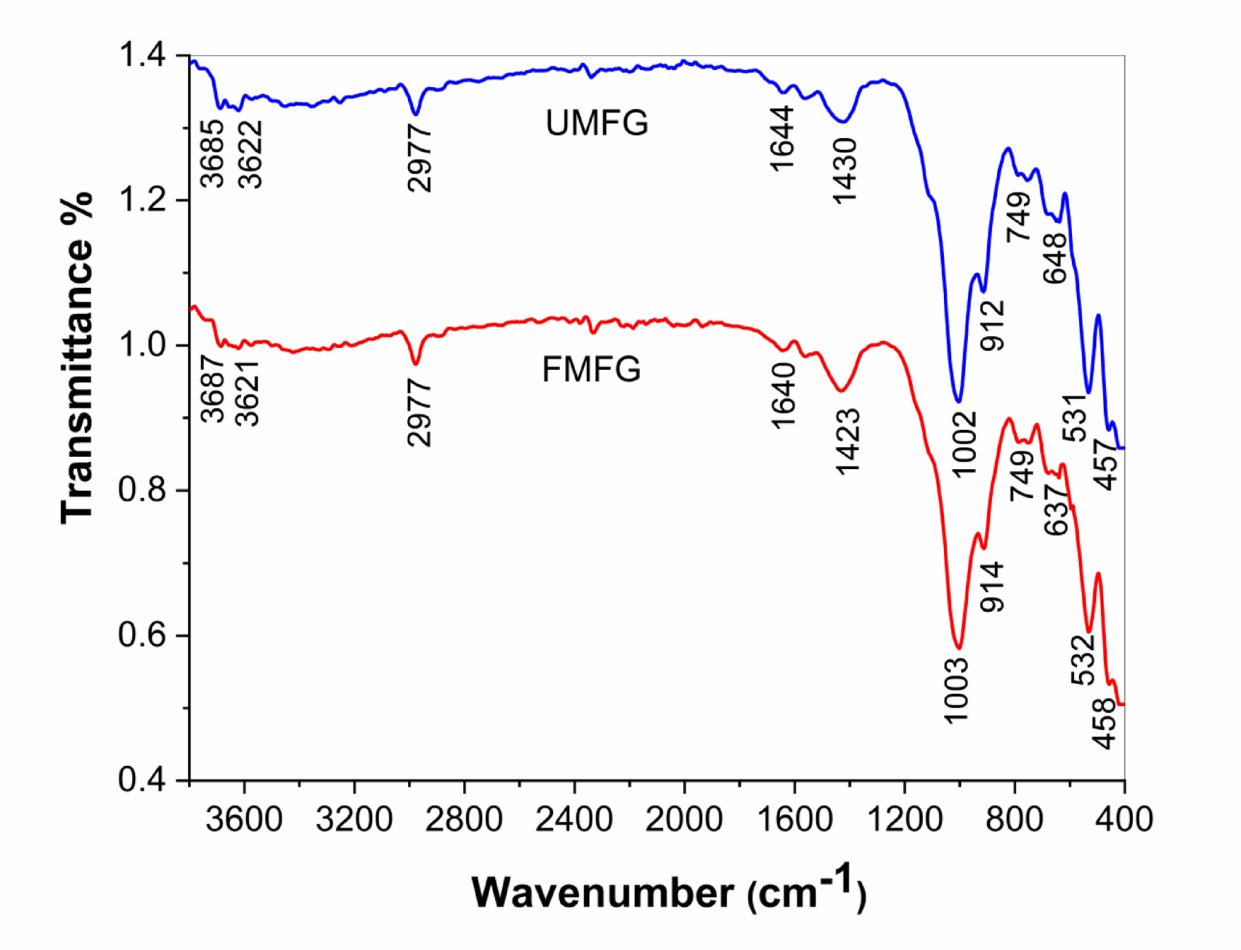
IR spectra of UMFG and FMFG after 28 days of hydration.

Figure 4 displays the infrared spectra of the geopolymer composites UMFG and FMFG prior to metal adsorption. There was no discernible variation in the IR spectra for both adsorbates, which showed typical aluminosilicate vibrations. Each sample exhibited a distinct absorption band at 1002 cm^−1^ owing to the Si-O-T (T = Si or Al) asymmetric stretching vibration. This band shifted to a lower wavenumber and gained a higher sharpness when compared with the pure MK (see Fig. 2), indicating that SiO_4_ tetrahedra was substituted by AlO_4_ to form the 3D network structure^39^. The resonance at 531 cm^−1^is assigned to the elongation vibration of Si–O–Al^17^^[,34^. Whereas the broad bands within the range between 3621 and 3687 cm^−1^ and around 1640 and 1644 cm^−1^ were attributed to stretching and deformation vibrations of the O–H and H–O–H groups from the bound water molecules, respectively^39^. In addition, the bands observed at 1423–1430 cm^−1^ are assigned to the asymmetric C-O stretching and out-of-plan C-O bending modes respectively, implying the presence of carbonation by atmospheric CO_2_. Finally, the band appears at 637–749 cm^−1^ is attributed to asymmetric bending of Si–O–Si/Si–O–Al and Si–O–Al bonds.

#### X-ray diffraction (XRD)

**Fig. 5:**
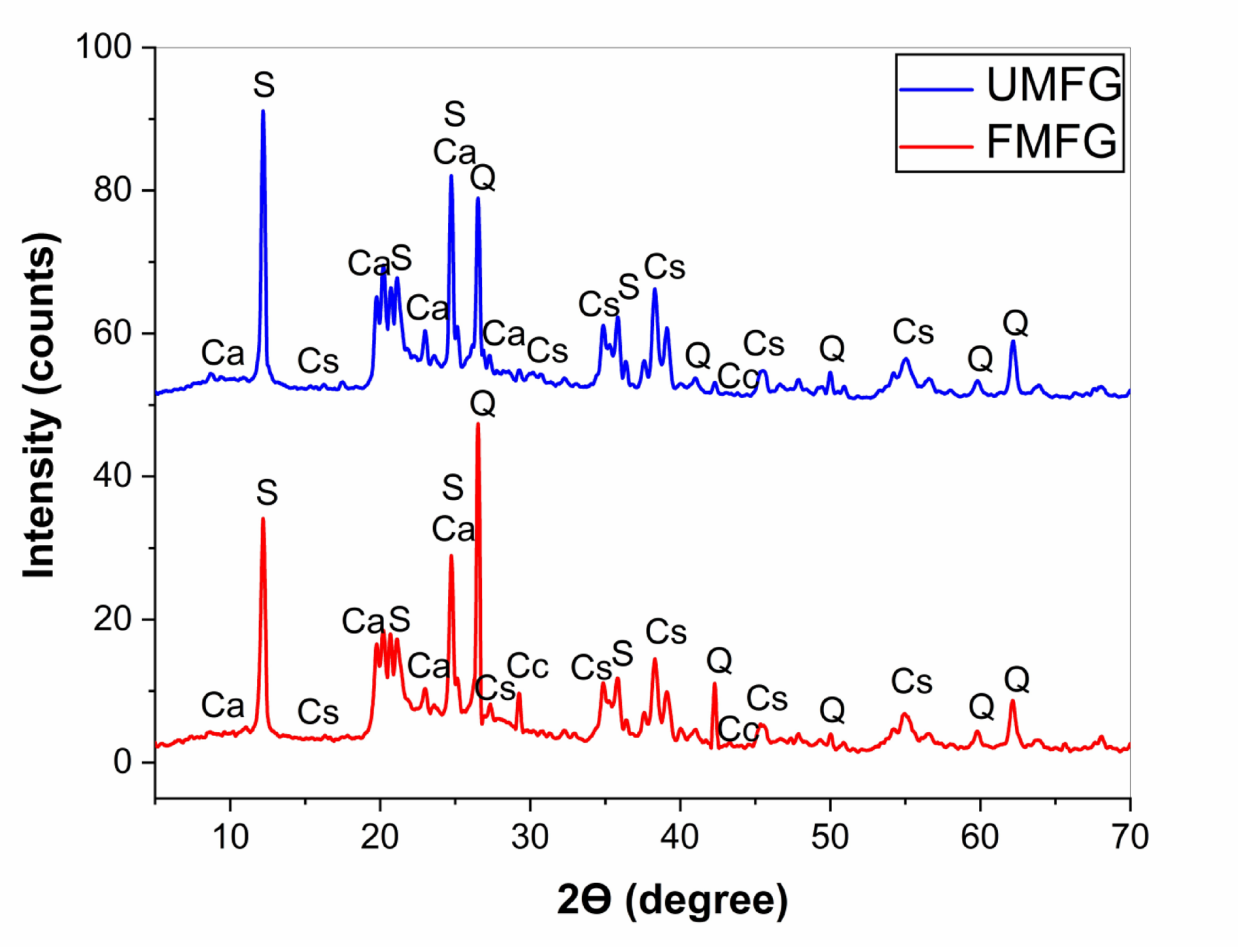
XRD pattern of foamed and un-foamed geopolymer paste after 28 days of hydration (Ca: C-A-S-H, Cs: C-S-H, S: N-A-S-H, Cc: Calcite, and Q: Quartz).

Figure 5 shows the XRD diagrams of the FMFG and UMFG pastes cured under relative humidity 100% for up to 28 days. Notably, both samples display the diffuse hump at angle (20^o^−35^o^ 2θ) consistent with an amorphous C-S-H [PDF 00–011–0304, PDF 00–011–0507], C-A-S-H [PDF//00–024–0181] and N-A-S-H aluminosilicate gel [PDF//00–013–0129, PDF//00–055–1018], which was the main responsible for the mechanical properties of metakaolin geopolymer^40^. Most of the kaolinite peaks vanished as a result of dissolution in alkali solution. The dominant crystalline peaks represented the quartz (SiO_2_) [PDF//01–089–1961].These minerals that were originally present in the raw materials are still observed after the geopolymerization process, which confirms the low solubility of the crystalline phases by the activating solutions. In addition, the hydrogen peroxide did not alter the XRD pattern of foam geopolymer^41^.

#### Pore structure distribution analysis

**Fig. 6:**
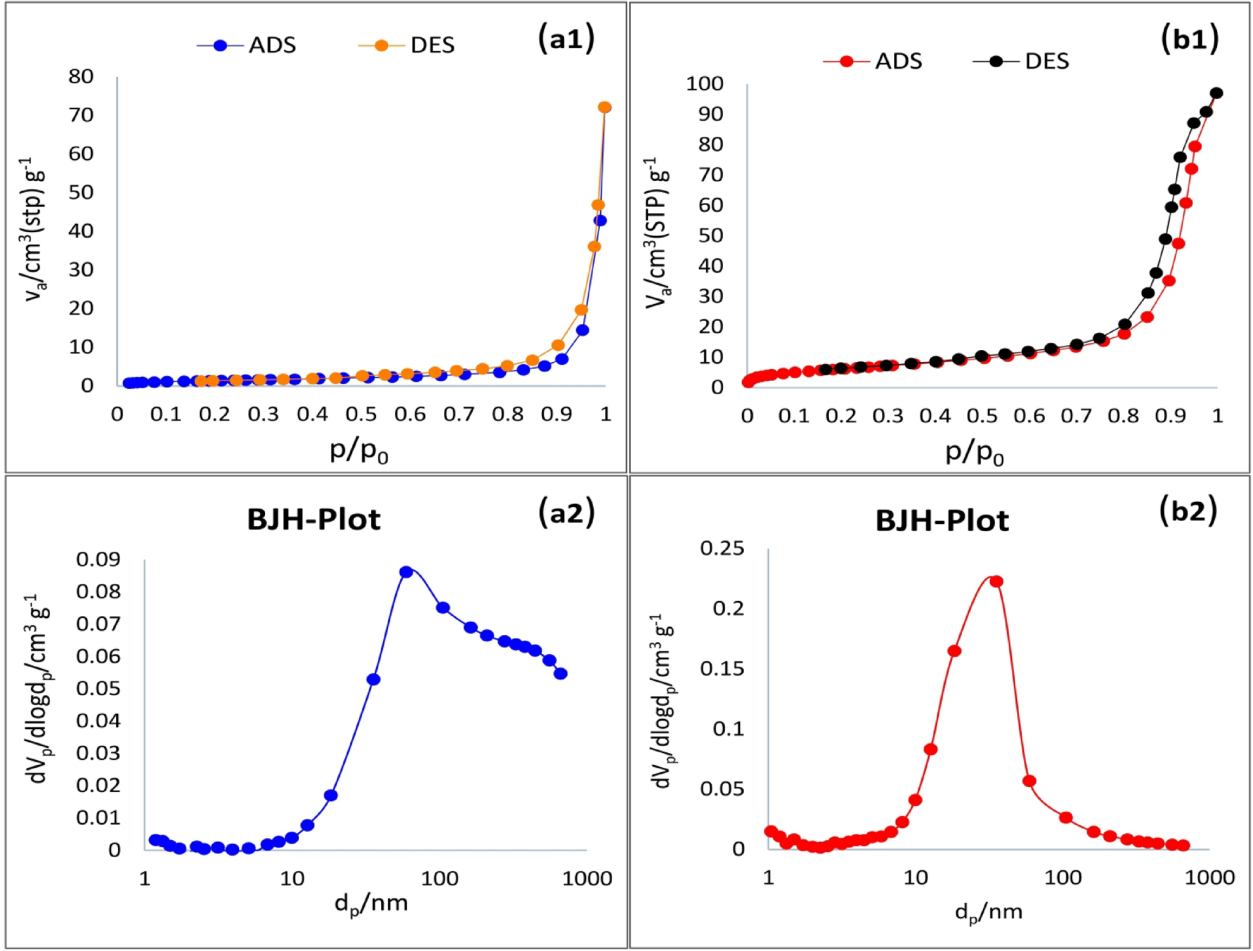
(a1-b1) N_2_-adsorption/desorption isotherm and (a2-b2) BJH- pore size distribution for UMFG and FMFG, respectively.

As shown in Fig. 6, N_2_ adsorption–desorption isotherms reveal the pore size distribution and specific surface area of the non-foamed (UMFG) and porous geopolymer (FMFG) adsorbents. As illustrated in the isotherms of Fig. 6b, the adsorption isotherms of the FMFG sample fit type IV characteristics with an H3 hysteresis loop, according to the (IUPAC) classification, suggesting the capillary condensation and the existence of mesopores (pores in the range of 2–50 nm)^42^. The BET surface area, total pore volume, and average pore diameter of both samples are tabulated in Table 2. The porous geopolymer (FMFG) exhibited a BET surface area and total pore volume of 23.052 m^2^/g and 0.1454 cm^3^/g, respectively. In contrast, UMFG displayed a lower specific surface area (5.1159 m^2^/g) and total pore volume (0.0722 cm^3^/g). The greater increase in the specific surface area and total pore volume of the FMFG sample compared to UMFG can be attributed to the foaming effect of H_2_O_2_ that induced pores in the structure, which may provide a more organized structure with an open frame structure during the polymerization process^37^. Notably, the average pore diameter of the FMFG (12.61 nm) was lower than that of UMFG (28.23 nm), suggesting the presence of a uniform pore structure.This finding was similar to the results of Xianyao et al. ^43^, who revealed that the structure of geopolymers was regulated by H_2_O_2_. The higher surface area of FMFG is related to the small sized mesopores and the higher pore volume compared to UMFG. This is consistent with the hypothesis that UMFG has a compact texture with less porosity was further supported by its smaller pore volume, as well as the physical characteristics as discussed in next section.

**Table2:** The N_2_-adsorption characteristic parameters of foamed and un-foamed geopolymers.

Adsorbents	Specific surface area (m^2^/g)	Total pore volume (cm^3^/g)	Average pore diameter (nm)
UMFG	5.1159	0.0722	28.231
FMFG	23.052	0.1454	12.615

#### Physio-chemical and mechanical characteristics

**Fig. 7:**
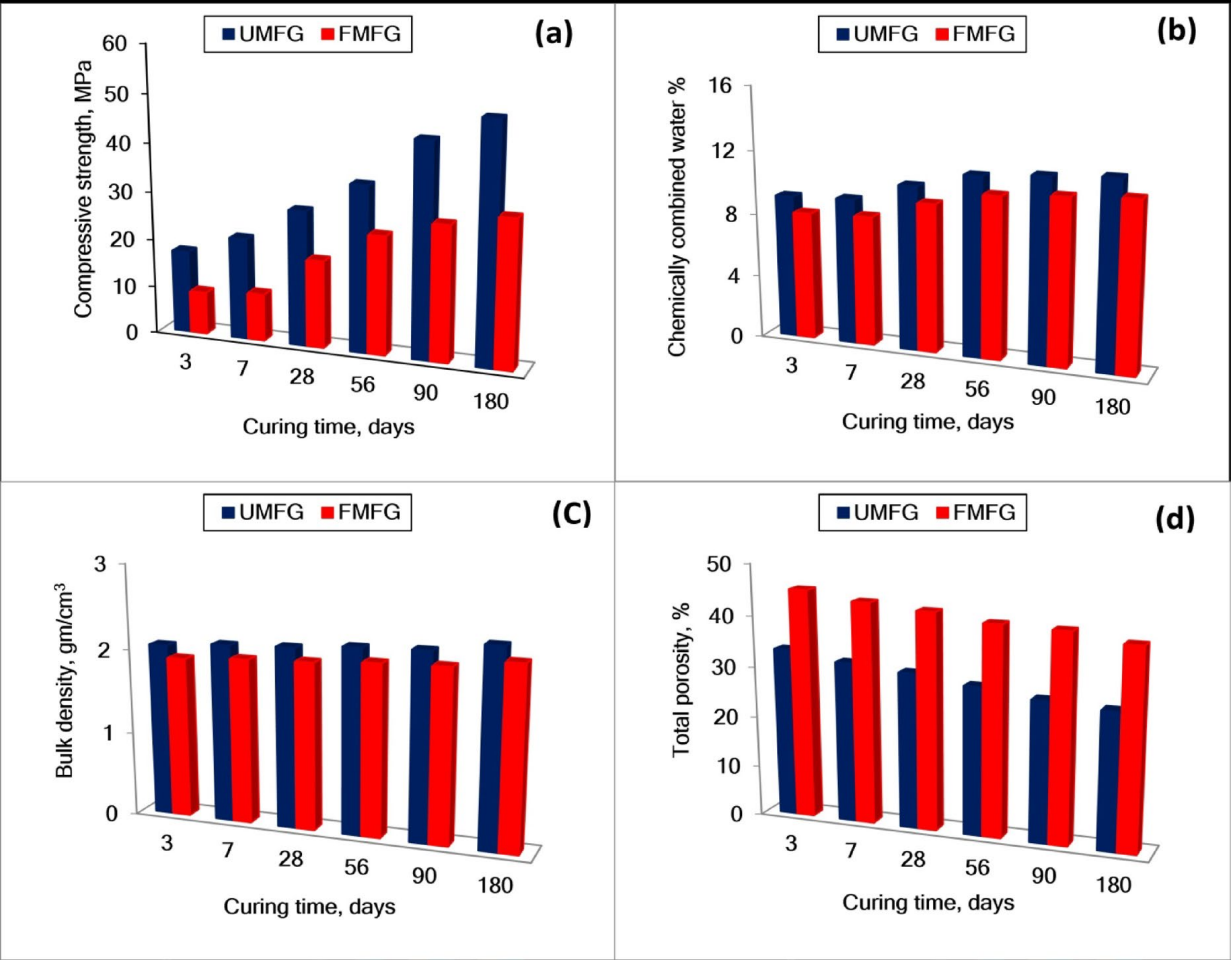
Physico-chemical and mechanical properties of alkali-activated MK-FA at different times (**a**) compressive strength (**b**) chemically combined water (**c**) bulk density (**d**) total porosity.

The compressive strengths of UMFG and FMFG cured under relative humidity 100% up to 180 days are plotted in Fig. 7. It is noticed that as the curing time increases, the compressive strength of both foamed and un-foamed geopolymer specimens increases. This is due to the sustainable hydration and assemblage of the hydrated products, which precipitated in the available pores as the time of the hydration increased^42^. However, the FMFG specimens appear to have comparatively lower values of compressive strength than the UMFG at most of all curing ages of the hydration process. This is related to the increased pores generated by the decomposition of H_2_O_2_, which increases the total porosity, implying the decreases in the compressive strength^40^.The total porosity values are in good agreement with the compressive strength values. Results showed a clear increase in the porosity of the foamed geopolymer specimens compared with the control specimens. This is due to hydrogen peroxide being thermodynamically unstable and can be easily decomposed to water and oxygen bubbles trapped within the paste, expanding and increasing the volume^44^. It is observed that the content of combined water as well as the bulk density of the alkali-activated MK-FA pastes increase gradually up to 180 days. This mainly attributed to sustainable activations and the formation of hydrated products. These hydrated products are deposited in the available open pores, which enhances the combined water content and also the bulk density of the geopolymer specimens. The addition of the foaming agent H_2_O_2_ resulted in increased porosity, while the bulk density and compressive strength decreased^42^.

#### Point of zero charge (pH_pzc_)

**Fig. 8:**
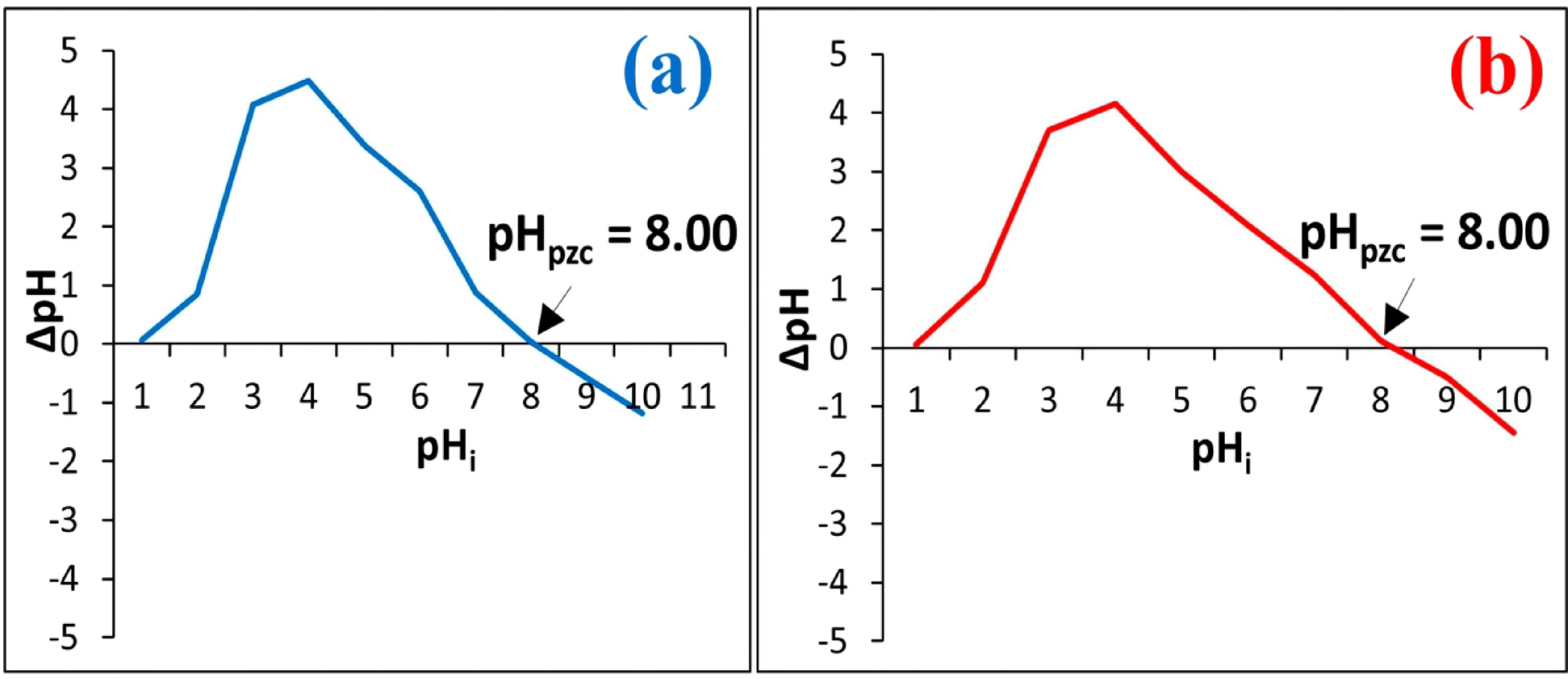
Point of Zero Charge for (**a**) UMFG and (**b**) FMFG.

Figure 8 displays the pHpzc of the foamed and non-foamed geopolymers. The results indicated that both geoadsorbents have the same pH_pzc_, demonstrating that the hydrogen peroxide added during the manufacturing process did not affect the surface functional groups. The point of zero charge (PZC) of both geoadsorbents was found to be at pH = 8.0, indicating an amphoteric surface, which is positively charged at pH lower than 8.0 and negatively charged when pH is higher than 8.0. Below the pH_pzc_ zero charge point, positive charges dominate on the surface, and the adsorbed amount of heavy metals in geopolymer decreases as a result of electrostatic repulsions between the positively charged heavy metals and the surface of these positively charged adsorbents. Also, the competition between heavy metals and the H^+^ ions, which are more mobile and will reach the geomaterials surface faster than heavy metals^45^. In contrast, at a higher pH, it is conceivable that the adsorption efficiency becomes higher due to the geoadsorbents carrying a net negative surface charge favors the adsorption of cationic heavy metals.

### Batch adsorption

#### Effect of pH

**Fig. 9:**
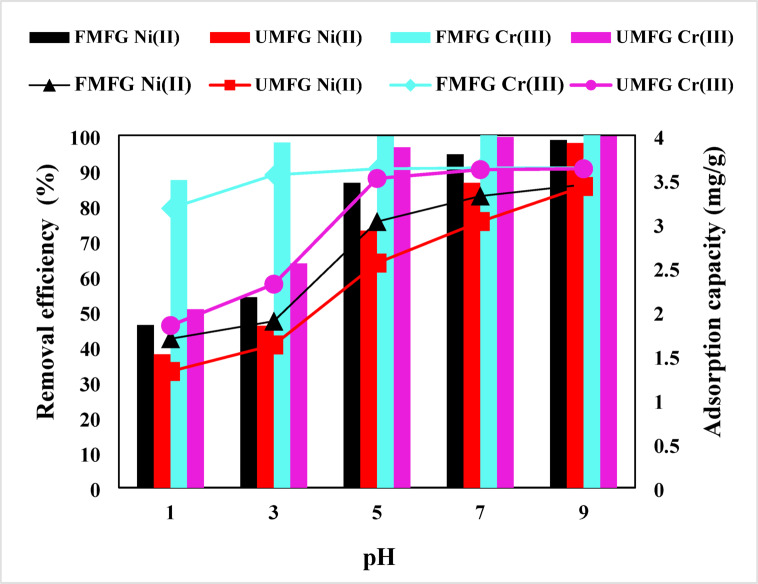
Effect of pH (volume: 100 mL, conc: 5 mg/L, dose: 0.1 g, time: 120 and 180 min. for Ni(II) and Cr(III), respectively).

The influence of pH on the sorption of heavy metals by geopolymers was studied from 1 to 9; other parameters: the initial concentration of Ni(II) and Cr(III) was 5.0 mg/L, and UMFG and FMFG were 0.1 g. The results obtained for different pH are presented in Fig. 9. When the pH value was increased from 1.0 to 5.0, a considerable jump in the removal efficiency of the UMFG and FMFG was observed to reach 72.94% and 86.48% for Ni(II) and 99.48% and 99.74% for Cr(III), respectively. Previous investigations have shown that at low pH (*<* 4), there is an excess of H^+^ ions surrounding the surface of the geopolymer that compete with the Ni(II) and Cr(III) ions for the available adsorption sites^46^. This trend is consistent across both adsorbents. Nonetheless, the porous geopolymer FMFG had a better adsorption capacity towards metallic species than the comparable geopolymer UMFG, which can be attributed to the increased specific surface area caused by the addition of H_2_O_2_. As the pH increases, the competition between the protons and Ni(II) or Cr(III) for active surface sites of geoadsorbents will decrease, and Ni(II) and Cr(III) ions are the predominating species^34^. Additionally, there was a notable removal of the target heavy metals at higher pH values and after the zero point of charge, which is attributed to the tendency of heavy metals to form insoluble micro-precipitates such as Ni(OH)_2_ and Cr(OH)_3_. The precipitation of Cr metals begins at a pH of solution exceeding 5.5 and may be resulting in a sorption distortion towards the two geopolymer materials Sari Ahmet et al. ^47^. According to Jiahong Wang et al. ^48^, Cr(III) precipitates at above pH 5.0. Kamel Al-Zboon et al. ^31^ found that the maximum adsorption of Cr^+3^ on volcanic tuff-based geopolymer occurred at pH 5.0; however, it decreases with a further increase in pH due to chromium hydroxide starting to precipitate, making true adsorption studies impossible. Yasser Hannachi et al. ^49^ used pH 5 as the optimum for Ni^+2^ uptake on calcined phosphate. They found that at higher pH values (pH 6–11), hydroxyl ions compete for Ni(II) with the active sites, leading to precipitation of nickel hydroxyl on the surface of the adsorbents. A similar finding was also reported by Hem Lata et al. ^6^. They sequestered Ni^+2^ on activated carbon and selected the optimal pH to be 5.0 because they found that when pH is increased, Ni(II) ions undergo precipitation through their interaction with hydroxide ions (OH⁻), leading to the formation of insoluble nickel hydroxide [Ni(OH)₂]. Therefore,the pH value of 5.0 was selected as the optimal pH for more adsorption experiments.

#### Effect of contact time

**Fig. 10:**
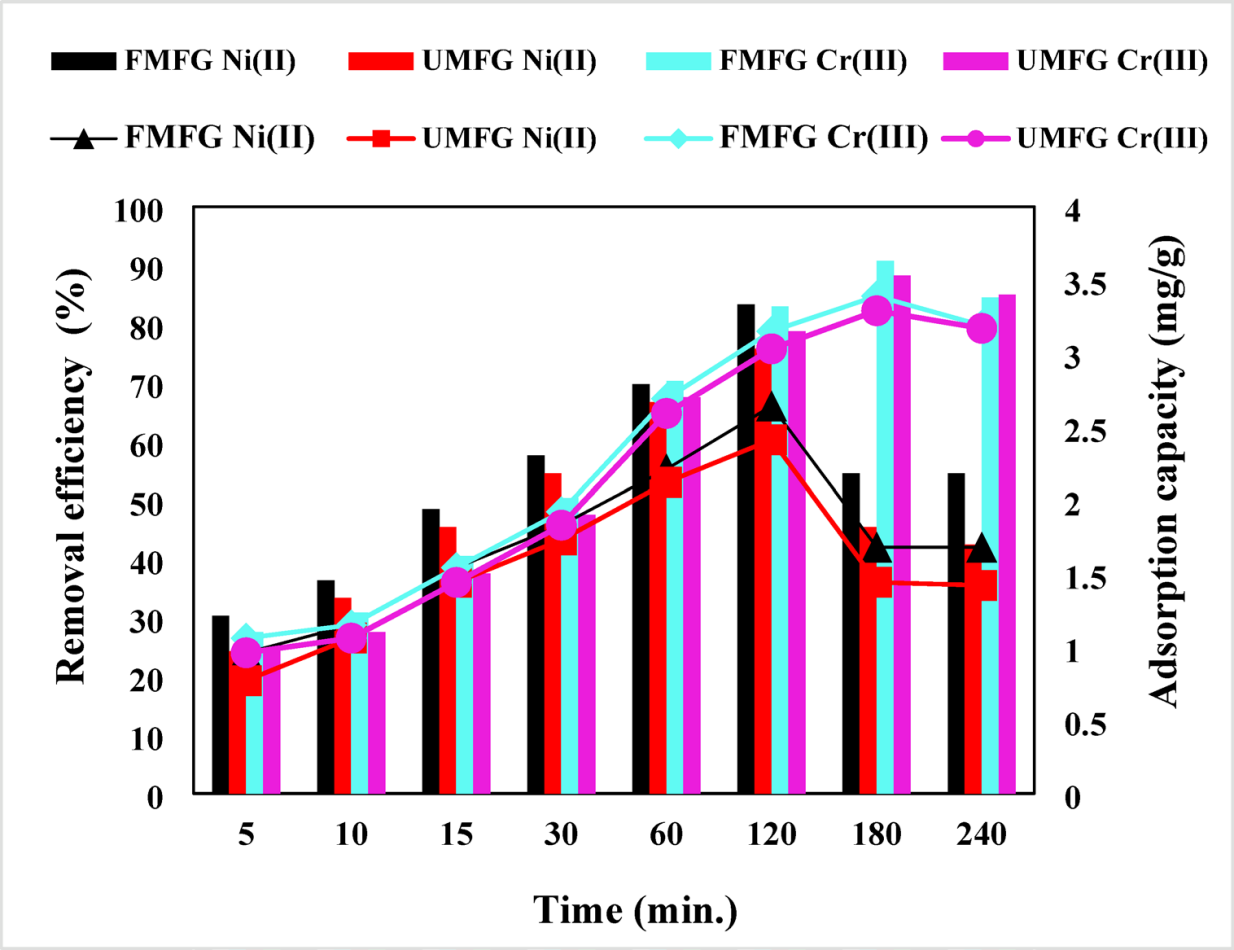
Effect of contact time (volume: 100 mL, pH: 5, conc: 5 mg/L, dose: 0.1 g).

The influence of contact time on the removal of Ni(II) and Cr(III) ions from an aqueous phase with an initial concentration of 5.0 mg/L for the UMFG and FMFG adsorbents was investigated at different time intervals in the range of 5 to 240 min, as shown in Fig. 10. The results showed that the expeditious adsorption of Ni(II) and Cr(III) ions occurred in the first 60 min due to a huge number of vacant adsorption sites on the adsorbent surface. It can be seen that the adsorption of both metal ions on geoadsorbents increases with an increase of contact time, and the equilibrium time is reached within 120 and 180 min for Ni(II) and Cr(III), respectively; afterward it remains constant (plateau). This is because a longer contact time between the metal solutions and the sorbent surface results in more adsorption by activating the equilibrium^36,37^. It is remarkable that 75.76% of Ni(II) and 88.25% of Cr(III) ions are adsorbed at equilibrium contact time for UMFG and 83.33% and 90.75% for FMFG, respectively. The higher adsorption of heavy metal ions in the porous geopolymer is perhaps due to the higher availability of active sites where the ions can penetrate it easily^36^.

#### Effect of dosage

**Fig. 11:**
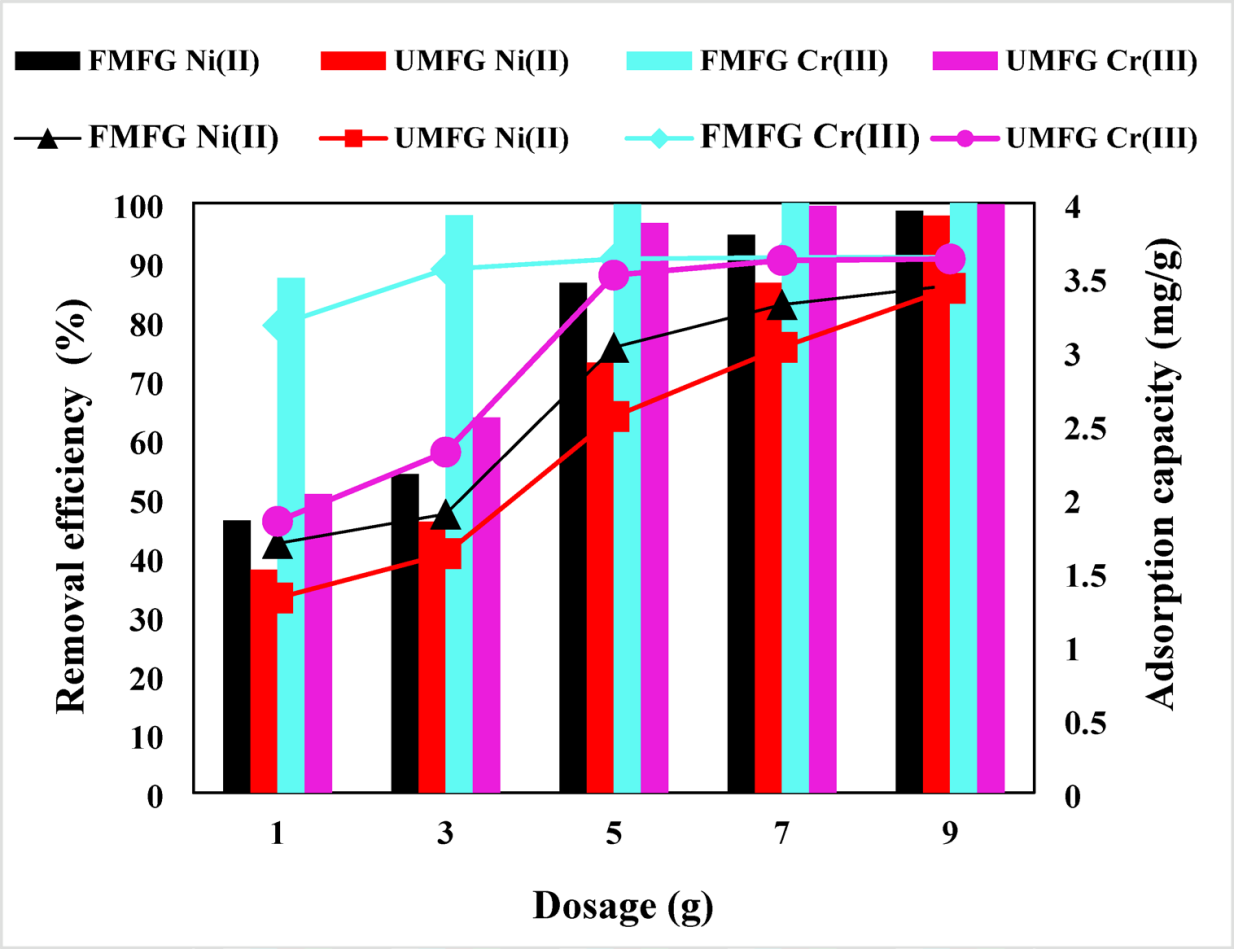
Effect of dosage (volume: 100 mL, pH: 5, conc: 5 mg/L, time: 120 and 180 min. for Ni(II) and Cr(III), respectively).

The influence of adsorbent dose on the removal of Ni(II) and Cr(III) ions was investigated, and the results are shown in Fig. 11. The experiments were conducted with various sorbent masses of 0.02, 0.04, 0.06, 0.08, 0.1, and 0.2 g per 100 mL of aqueous solution of both ions. The results revealed that as the adsorbent mass increased from 0.02 to 0.2 g, the removal efficiency increased dramatically from 63.88% to 81.31% for Ni(II) and from 88.62% to 98.44% for Cr(III) by UMFG, while the removal efficiency was boosted by the FMFG from 72.22% to 91.66% and from 94.13% to 99.65% for Ni(II) and Cr(III), respectively. As expected, the removal efficiency of the porous FMFG materials has higher values than conventional UMFG materials; this could be due to the increased adsorbent surface area, pore volume, and the greater availability of the exchangeable sites for interaction with Ni(II) and Cr(III) ions^26^. Generally, the removal efficiency of heavy metals is dependent on the available binding sites on the adsorbent^40^. On the other hand, the uptake capacity of both adsorbents was decreased because the adsorption active sites of adsorbent available was not fully utilized at a higher adsorbent dosage in comparison to a lower adsorbent dosage. Therefore, the optimal dosage in this work is 0.1 g to avoid ineffective overdose.

#### Effect of initial ions concentration

**Fig. 12:**
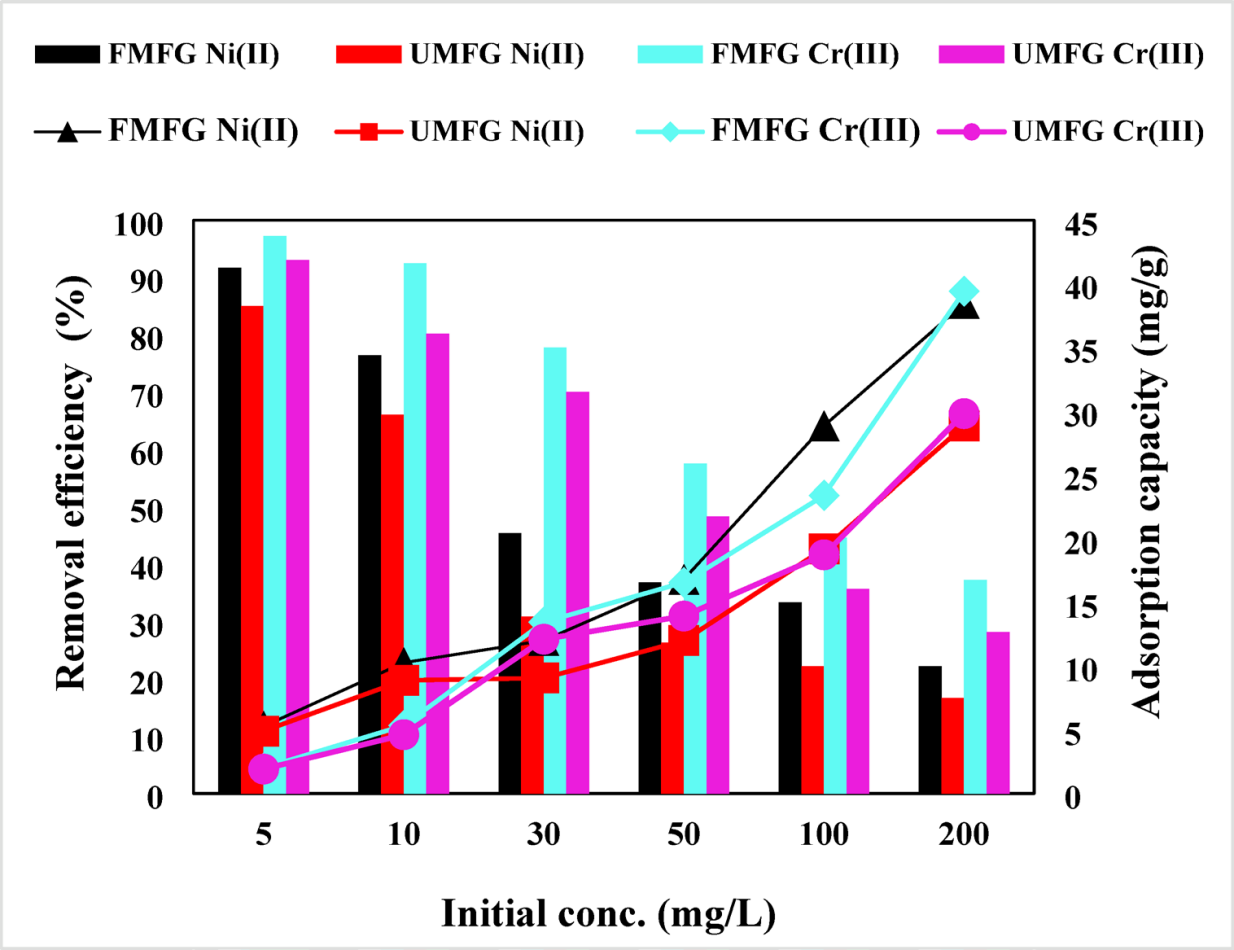
Effect of initial concentration (volume: 100 mL, pH: 5, dose: 0.1 g, time: 120 and 180 min. for Ni(II) and Cr (III), respectively).

The effect of varying concentrations of Ni(II) and Cr(III) on the adsorption capacity is ascertained in Fig. 12. The batch experiment runs were conducted using different metal concentrations: 5, 10, 30, 50, 100, and 200 mg/l, with a sorbent dose of 0.1 g. Where it is evident that the adsorption efficiency decreased with increasing initial metal ion concentration from (5–200 mg/l), this can be attributed to the deficiency of sufficient vacant binding sites^33,38^. As a result, the effectiveness was decreased by the FMFG from 91.67% to 22.22% and from 97.21% to 37.27% for Ni(II) and Cr(III), respectively. While the UMFG reduced remarkably the effectiveness from 85.00% to 16.67% and from 93.02% to 28.18% for Ni(II) and Cr(III), respectively. A similar trend in results was also observed by Tan et al. ^46^. At lower concentrations, the available binding sites in the adsorbent surface were larger and not fully utilized, resulting in an enhanced sorption capacity. Once the initial metal concentration increases, the saturation of the adsorption active sites occurred; hence the ratio of the adsorbent to the adsorbate decreased.

The low sorbent-to-sorbate ratio indicates that there was no further binding site for adsorption. Nevertheless, based on the comparison, the Ni(II) and Cr(III) removal capacity of such a foamed geopolymer specimen is higher than that of a non-foamed geopolymer, suggesting that the porous geopolymer surface has a larger number of pores, which are sufficient to adsorb most of the metal ions^40^.

**Fig. 13:**
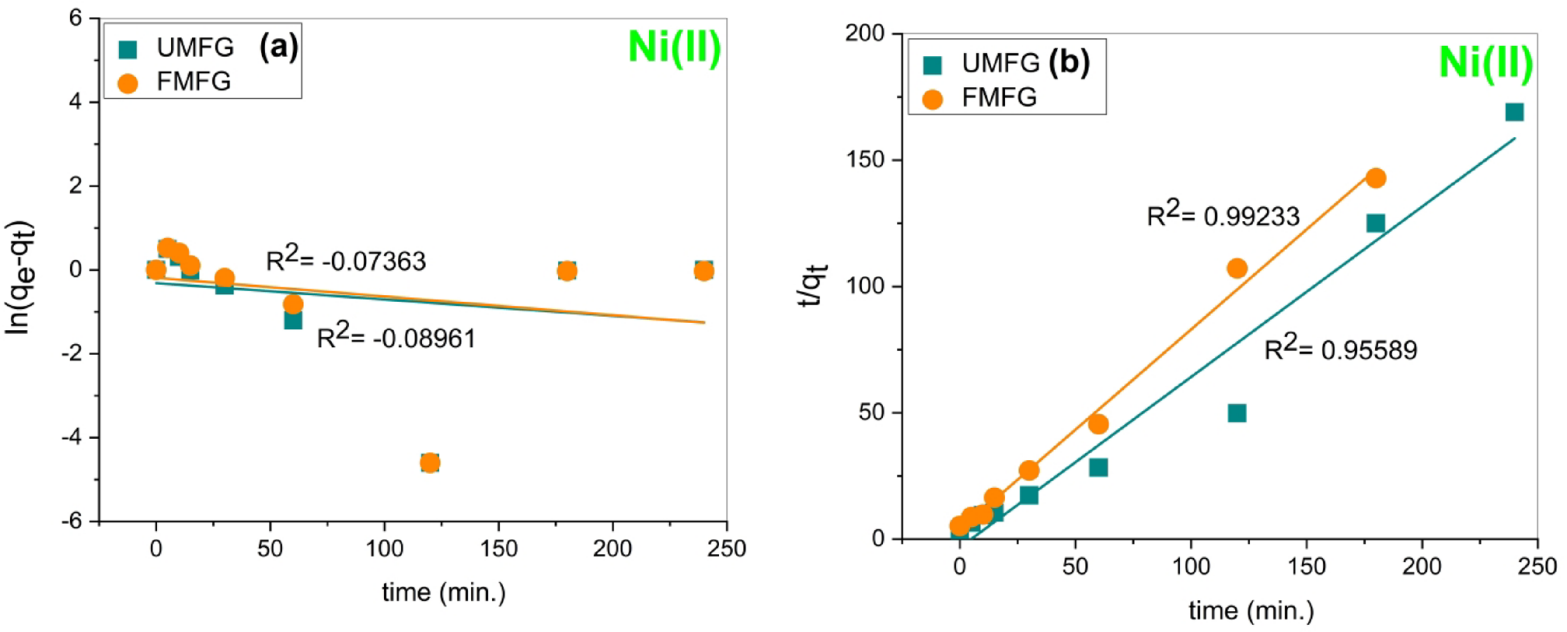
Kinetic model plots for Ni(II) adsorption using UMFG and FMFG: (**a**) pseudo-first-order and (**b**) pseudo-second-order (volume: 100 ml, pH: 5, dose: 0.1 g, conc: 5 mg/L).

#### Adsorption kinetics

**Fig. 14:**
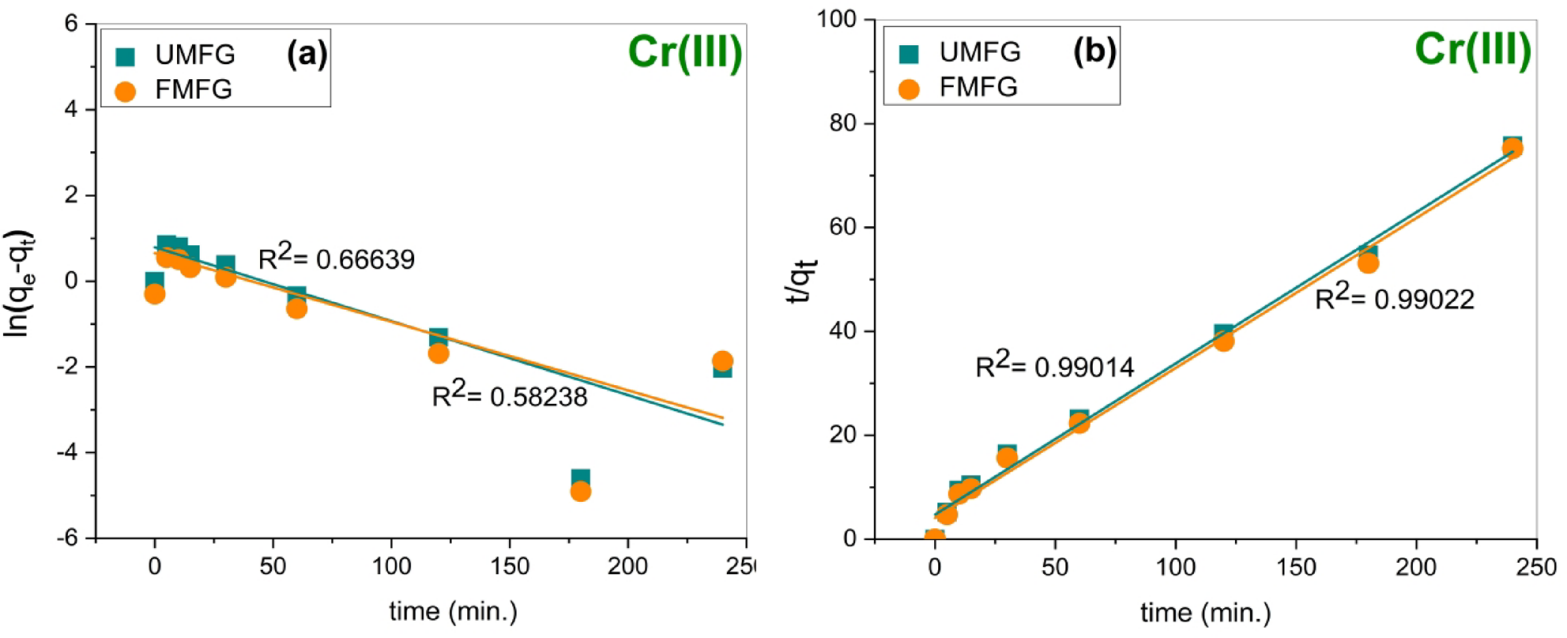
Kinetic model plots for Cr(III) adsorption using UMFG and FMFG: (**a**) pseudo-first-order and (**b**) pseudo-second-order (volume: 100 ml, pH: 5, dose: 0.1 g, conc: 5 mg/L).

Figure 10 shows that the Ni(II) and Cr(III) removal efficiency increases with contact time, which reveals that the adsorption is a dynamic process^33,44^. The adsorption kinetics of the foamed and un-foamed geopolymer specimens were investigated using pseudo-first-order and second-order kinetic models. The study of adsorption kinetics plays an essential role in elucidating the mechanism of adsorption. Figures 13 and 14 illustrate the fitting results of pseudo-first-order and pseudo-second order kinetics models, and the kinetic parameters are presented in Table 3. The correlation coefficient R^2^ of the pseudo-second-order model is higher than that of the pseudo-first-order model. This indicates that the experimental data fit more closely with the pseudo-second-order model, suggesting that the mechanism of adsorption for Ni(II) and Cr(III) was mainly chemisorption^46^.

**Table 3:** Kinetic parameters for Ni(II) and Cr(III) adsorption using foamed and un-foamed geopolymers.

Adsorbents	UMFG			FMFG	
Metal		Ni(II)	Cr(III)	Ni(II)	Cr(III)
Pseudo- first-order	q_e_ (exp) (mg.g^−1^)	2.41	3.29	2.64	3.39
	q_e_ (theo) (mg.g^−1^)	0.72963563	1.89486955	0.82712454	1.810910939
	K_1_ (min^−1^)	0.0039	0.01725	0.00446	0.01601
	R^2^	−0.08961	0.66639	−0.07363	0.58238
Pseudo- second-order	q_e_ (exp) (mg.g^−1^)	2.41	3.29	2.64	3.39
	q_e_ (theo) (mg.g^−1^)	1.48240386	3.43359428	1.26165453	3.46428324
	K_2_ (g.mg^−1^.min^−1^)	−0.51680534	0.35852482	0.45453078	0.41113919
	R^2^	0.95589	0.99014	0.99233	0.99022

#### Adsorption isotherms

**Fig. 15:**
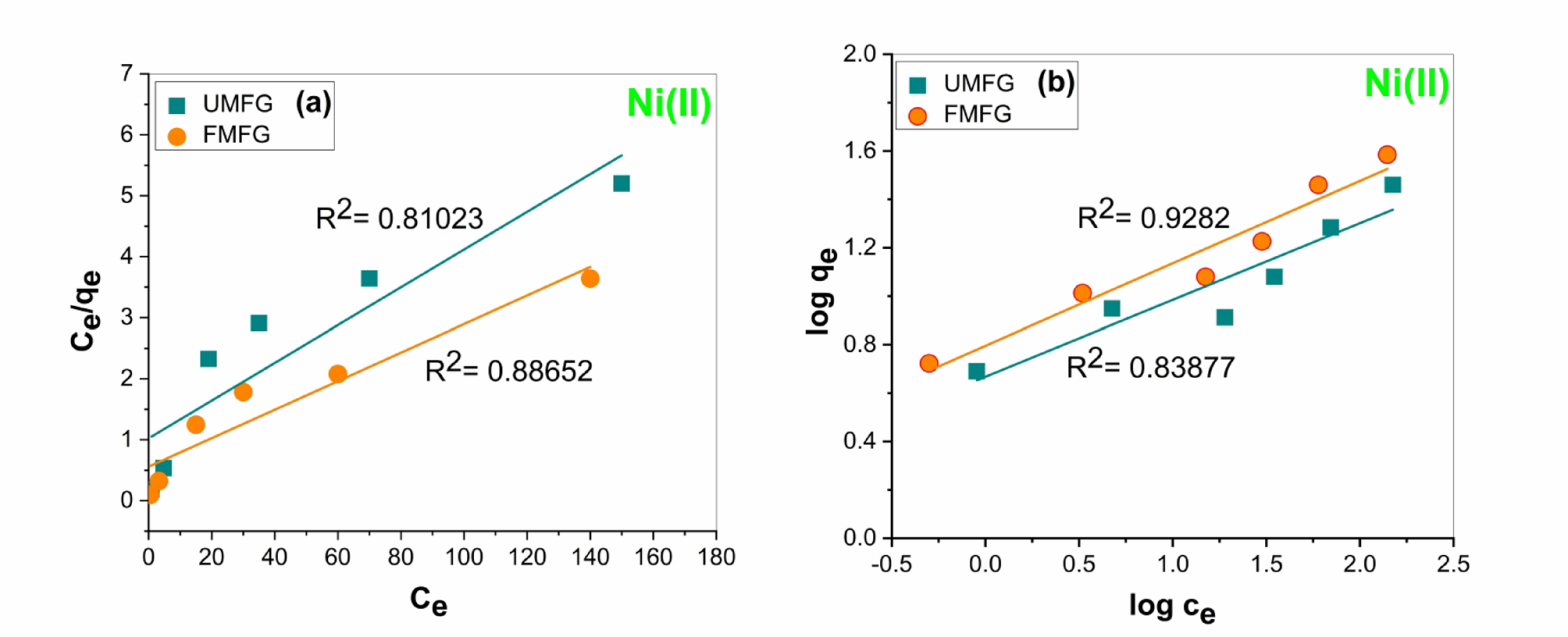
Isotherm model plots for Ni(II) adsorption using foamed and un-foamed MFG: (**a**) Langmuir and (**b**) Freundlich (volume: 100 mL, pH: 5, dose: 0.1 g, time: 120 min).

**Fig. 16:**
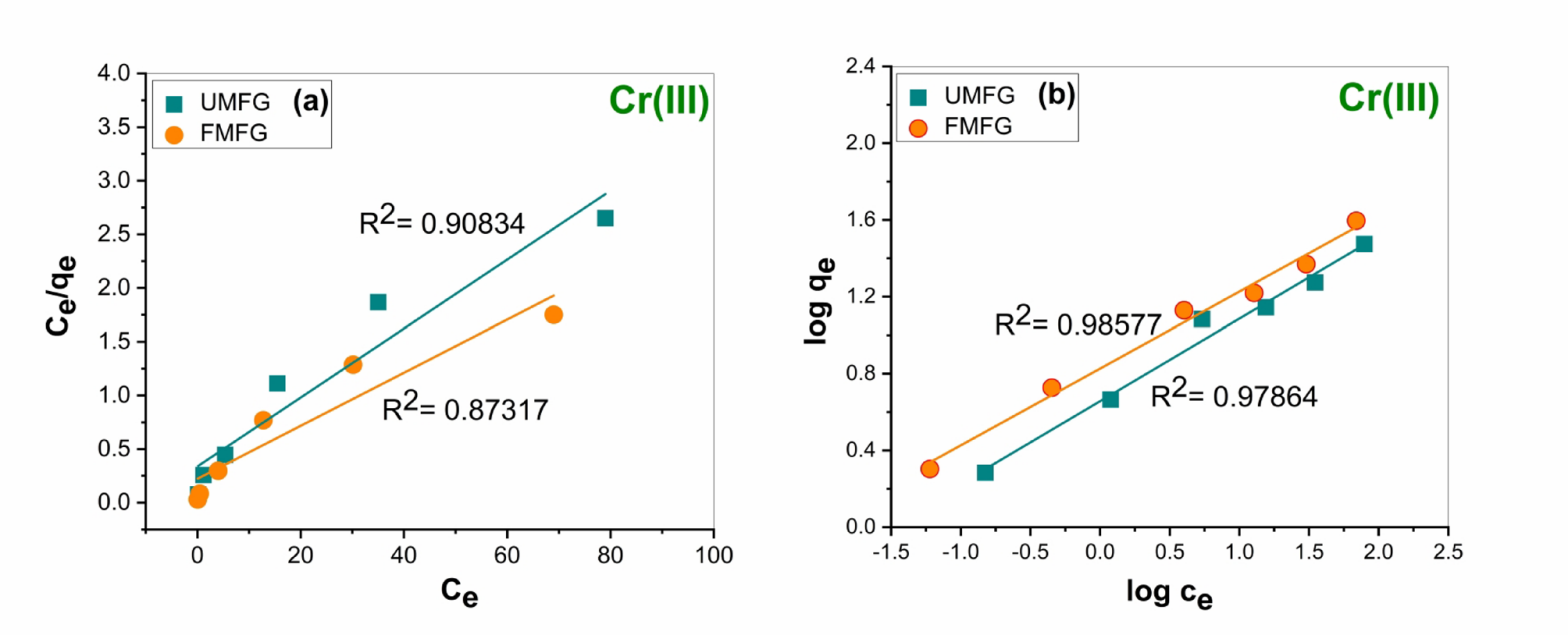
Isotherm model plots for Cr(III) adsorption using foamed and un-foamed MFG: (**a**) Langmuir and (**b**) Freundlich (volume: 100 mL, pH: 5, dose: 0.1 g, time: 180 min).

Figures 15 and 16 show the linear fitting of the experimental data using the Langmuir and Freundlich isotherm models. Moreover, Table 4 shows their respective fitting parameters. According to the correlation coefficient (R^2^) of the two examined isotherm systems, the Freundlich isotherm model showed a higher significant correlation (0.9282 and 0.98577) by FMFG compared to the Langmuir isotherm model (0.88652 and 0.87317) for Ni(II) and Cr(III), respectively. Although, UFMF show the correlation coefficients of Ni(II) and Cr(III) were 0.83877 and 0.97864, respectively, compared to the Langmuir isotherm model (0.81023 and 0.90834), indicating that adsorption behaviors could be fitted with a Freundlich isotherm model. In addition to that, the maximum theoretical Ni(II) and Cr(III) adsorption capacity values of both geopolymers anticipated by the Langmuir isotherm model were very far from their maximum experimental adsorption capacity, which was (28.34 mg/g and 29.81 mg/g) by UMFG and (38.46 mg/g and 39.42 mg/g) by FMFG for Ni(II) and Cr (III), respectively, compared with theoretical values as shown in Table 4. Also, the Freundlich isotherm constants are often exploited to assess their favourability for the adsorption process. When the value of n lies in the range of 1–10 (1 < *n* > 10), it is a favourable process that was successfully achieved as shown in Table 4, and the K_F_ value tends to the maximum adsorption capacity at the equilibrium concentration of adsorbate^37,38^. So, the calculated K_F_ values indicate that the adsorption capacity of FMFG 6.246547 and 6.713206 is greater than the UMFG 4.654682 and 4.539834 for Ni(II) and Cr(III), respectively. suggesting that the Freundlich model favoured well to describe the adsorption of Ni(II) and Cr(III) by both geoadsorbents.

**Table 4:** Isotherm parameters for Ni(II) and Cr(III) adsorption using foamed and un-foamed geopolymers.

Adsorbents	UMFG			FMFG	
Metals		Ni(II)	Cr(III)	Ni(II)	Cr(III)
Freundlich isotherm	K_F_ (L.mg^−1^)	4.654681828	4.539834281	6.246546827	6.713206401
	n	3.155868337	2.329102131	2.937979258	2.499937502
	R^2^	0.83877	0.97864	0.9282	0.98577
Langmuir isotherm	q_max_ (mg. g^−1^)	32.33107016	31.13325031	42.78990158	40.43671654
	K_L_ (L.mg^−1^)	0.031681599	0.010890286	0.013055417	0.005519736
	R^2^	0.81023	0.90834	0.88652	0.87317

#### Thermodynamics of adsorption

**Fig. 17:**
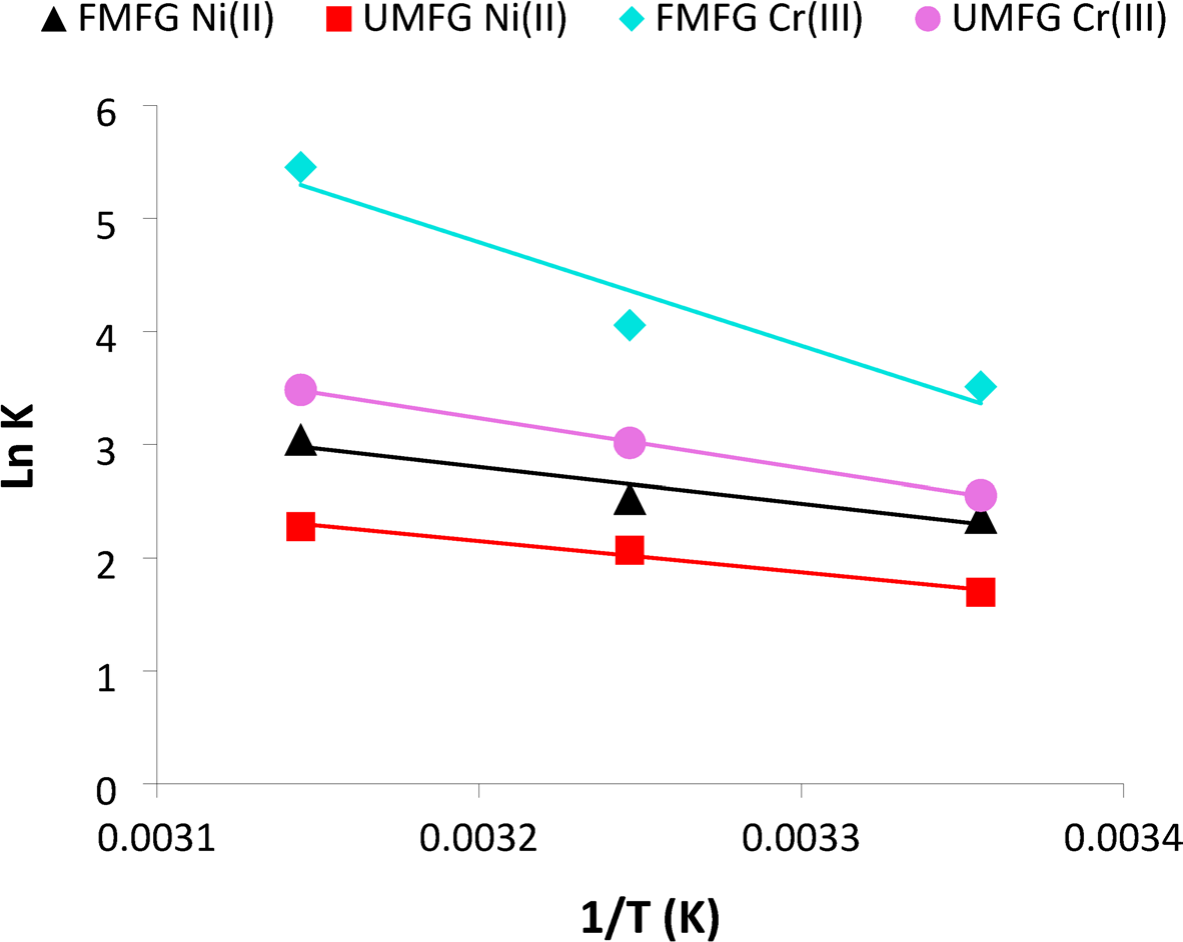
Plot of ln K against 1/T obtained for the adsorption of Ni^+2^ and Cr^+3^ onto UMFG and FMFG adsorbents.

The effect of temperature (25, 35, and 45 °C) on adsorption of Ni^+2^ and Cr^+3^ onto UMFG and FMFG adsorbents is shown in Fig. 17. Enthalpy change (ΔH) and entropy change (ΔS) can be obtained from slopes and intercepts of lnK versus 1/T plots. The calculated values of thermodynamic parameters of ΔH°, ΔS°, and ΔG° for Ni^+2^ and Cr^+3^ adsorption onto UMFG and FMFG are summarized in Table 5. The positive values of ΔH° indicate that the adsorption reaction is endothermic. According to (Jingmin Wan et al.^50^; Nuri and Ersoz^51^.), the adsorption process with ΔH° ranges from 20.9 to 418.4 kJ mol^−1^ and is recognized as a chemisorption process. The enthalpy values were found to be 22.939 and 36.694 kJ mol^−1^ for Ni(II) and Cr(III), respectively, when using UMFG adsorbent. Likewise, the ΔH° values changed to 26.862 and 76.129 kJ mol^−1^ for Ni^+2^ and Cr^+3^, respectively, by using FMFG. This confirmed that the chemisorption mechanism is involved during Ni^+2^ and Cr^+3^ adsorption on geopolymer. The entropy change (ΔS°) is positive, indicating a random increase during adsorption. These results corroborate the work of (Panda et al.^52^; Donat et al.^53^), which reported that when ΔH° and ΔS° values are positive, they suggest the adsorption to be chemisorption type. Moreover, positive values of entropy favor complexation and stability of sorption^53^, and also the reaction is irreversible^52^. The values of ΔG° determined are negative, thus confirming that the adsorption process is spontaneous and thermodynamically favorable^50^.

**Table 5:** Thermodynamic parameters calculated for Ni^+2^ and Cr^+3^ adsorption on foamed and un-foamed geopolymer.

Adsorbent	Adsorbate	∆H◦ (KJ/mol)	∆S◦ (J/mol. K)	∆G◦ (KJ/mol)		
				298 K	308 K	318 K
UMFG	Ni(II)	22.9399888	91.271092	−4.200426865	−5.298257496	−6.017680541
	Cr(III)	36.6946704	144.297784	−6.320401261	−7.71778743	−9.20834954
FMFG	Ni(II)	26.862534	109.254274	−5.843786046	−6.467663648	−8.049521577
	Cr(III)	76.1296352	283.432574	−8.699632525	−10.3840043	−14.41914772

### Characterization of geopolymer adsorbents after heavy metals adsorption

#### FT-IR patterns

**Fig. 18:**
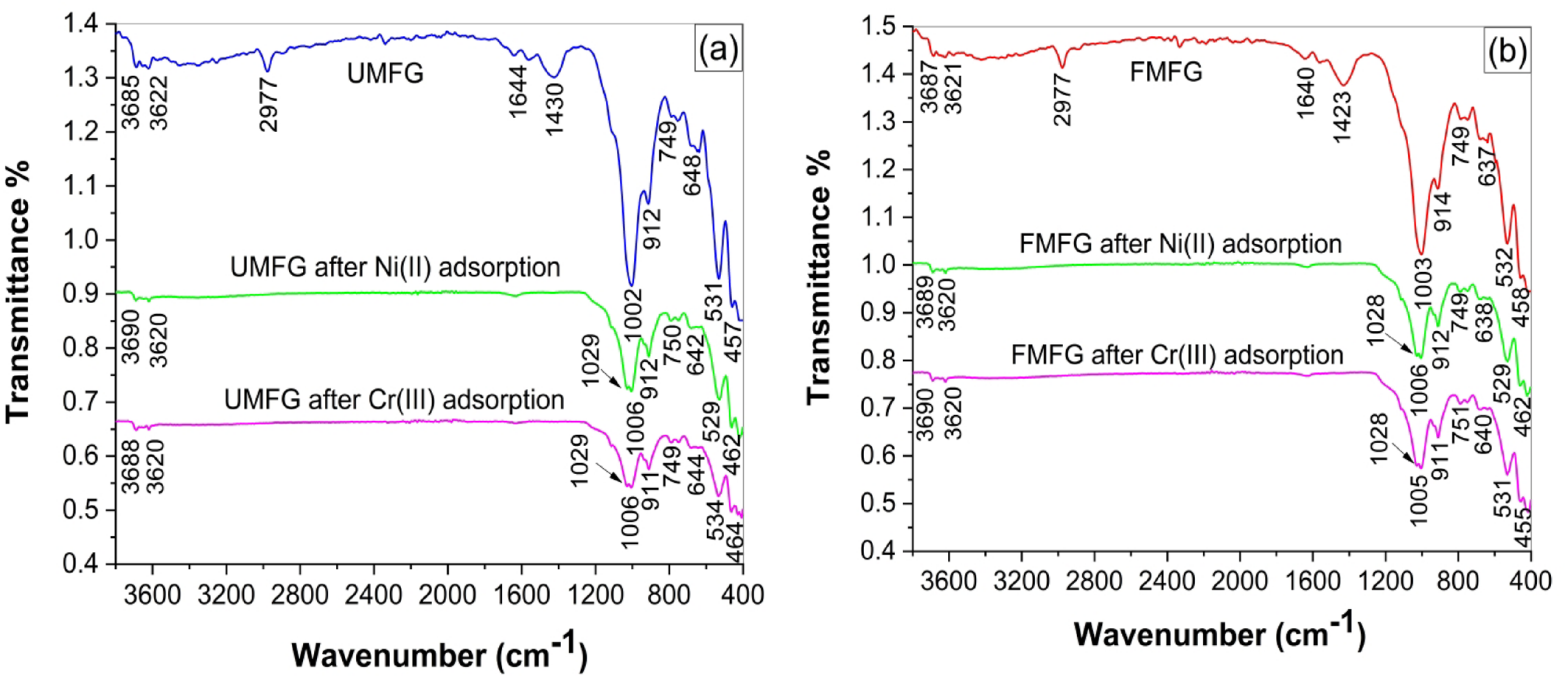
FT-IR spectra of (**a**) UMFG and (**b**) FMFG after Ni(II) and Cr(III) adsorption.

The FTIR spectra of the foamed and un-foamed geopolymers before and after Ni(II) and Cr(III) adsorption are shown in Fig. 18. The results revealed significant alteration after adsorption; some of the adsorption peaks are shifted or vanished, and new peaks are formed due to the adsorption of Ni(II) and Cr(III) ions onto the adsorbent surface. The peaks at 1002 cm^−1^ and 1003 cm^−1^ were ascribed to the Si-O-T (T = Si or Al) asymmetric stretching vibration, slightly shifted to 1006 cm^−1^ following Ni(II) and Cr(III) adsorption due to the interaction between heavy metal ions and the silicate of the adsorbent^34^. Meanwhile, a new peak was formed at 1028 and 1029 cm^−1^, implying Ni(II) and Cr(III) loading onto the adsorbent surface. Also, the absorption band of the hydroxyl functional group at 3622 cm^−1^ decreased compared to both geoadsorbents prior to adsorption, suggesting that the –OH group can undergo coordination reactions with metal ions during adsorption^47^. Changes in FT-IR spectra obtained before/after Ni(II) and Cr(III) adsorption onto the UMFG and FMFG adsorbents indicate that chemisorption could also be involved in the adsorption and also were considered as evidence for Ni(II) and Cr(III) adsorption.

#### SEM analysis

**Fig. 19:**
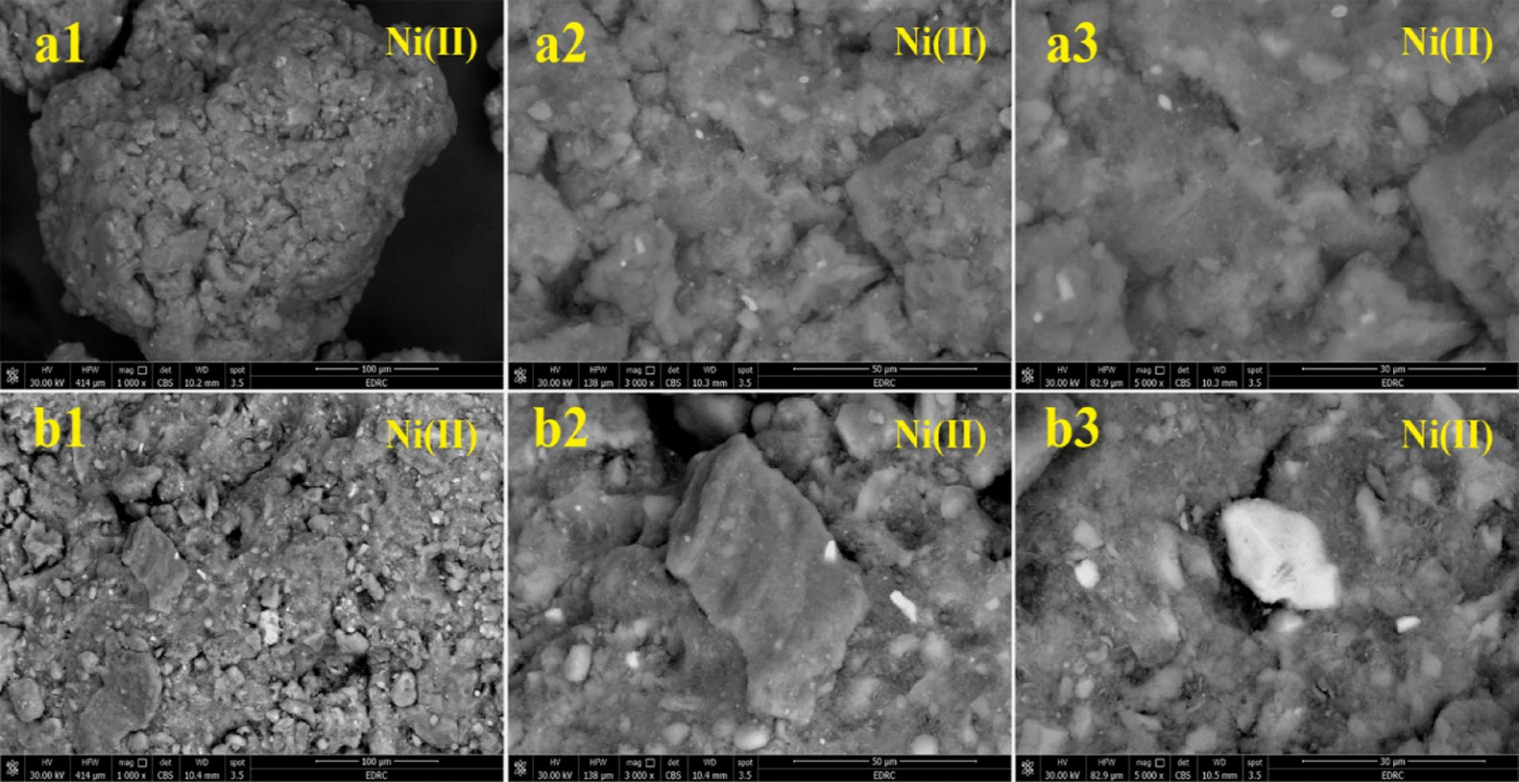
SEM image of (**a**) UMFG: (a1) 1000 x (a2) 3000 x (a3) 5000 x and (**b**) the FMFG: (b1) 1000 x (b2) 3000 x (b3) 5000 x after Ni(II) adsorption.

**Fig. 20:**
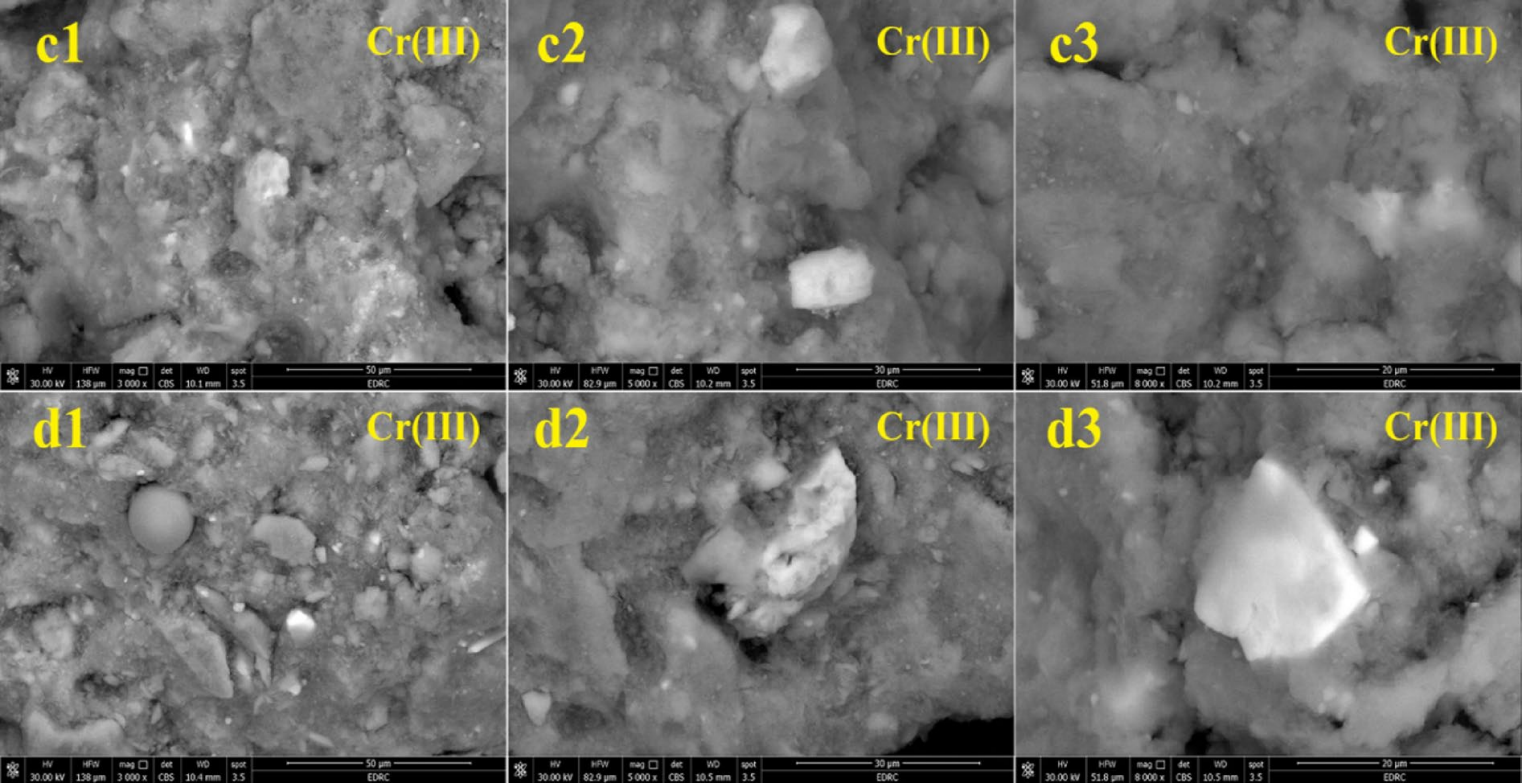
SEM image of (**a**) UMFG: (c1) 3000 x (c2) 5000 x (c3) 8000 x and (**b**) the FMFG: (d1) 3000 x (d2) 5000 x (d3) 8000 x after Cr(III) adsorption.

Figures 19 and 20 display the SEM images of UMFG and FMFG to verify the adsorption of Ni(II) and Cr(III) ions. It is evident from these figures that the surface morphology was completely changed before and after the adsorption of heavy metals. Figure 19 represents the SEM micrographs of both geoadsorbents after Ni(II) adsorption with different magnifications (from 1000X up to 5000X). In Fig. 19(a), the non-foamed geopolymer can be seen as white cloud-like particles, and Ni can be observed as white spots predominantly adsorbed on the surface of those geopolymer particles. While the SEM micrograph taken after the metal was loaded on the FMFG shows that the surface of the adsorbent was covered with Ni(II) metal ion, as can be seen in Fig. 19(b). It is impressive that the images of both geopolymers, which were taken at the same magnifications, revealed that the amount of Ni adsorbed by foamed geopolymer appears to be more than that of non-foamed geopolymer specimens^38,54^. Figure 20 indicates the surface texture of UMFG and FMFG following Cr(III) loading. It was observed that the surface of the adsorbents became smooth after the adsorption, which could be due to the entrapment of Cr(III) onto the sorbent after adsorption^37^.

### Adsorption mechanism

**Fig. 21:**
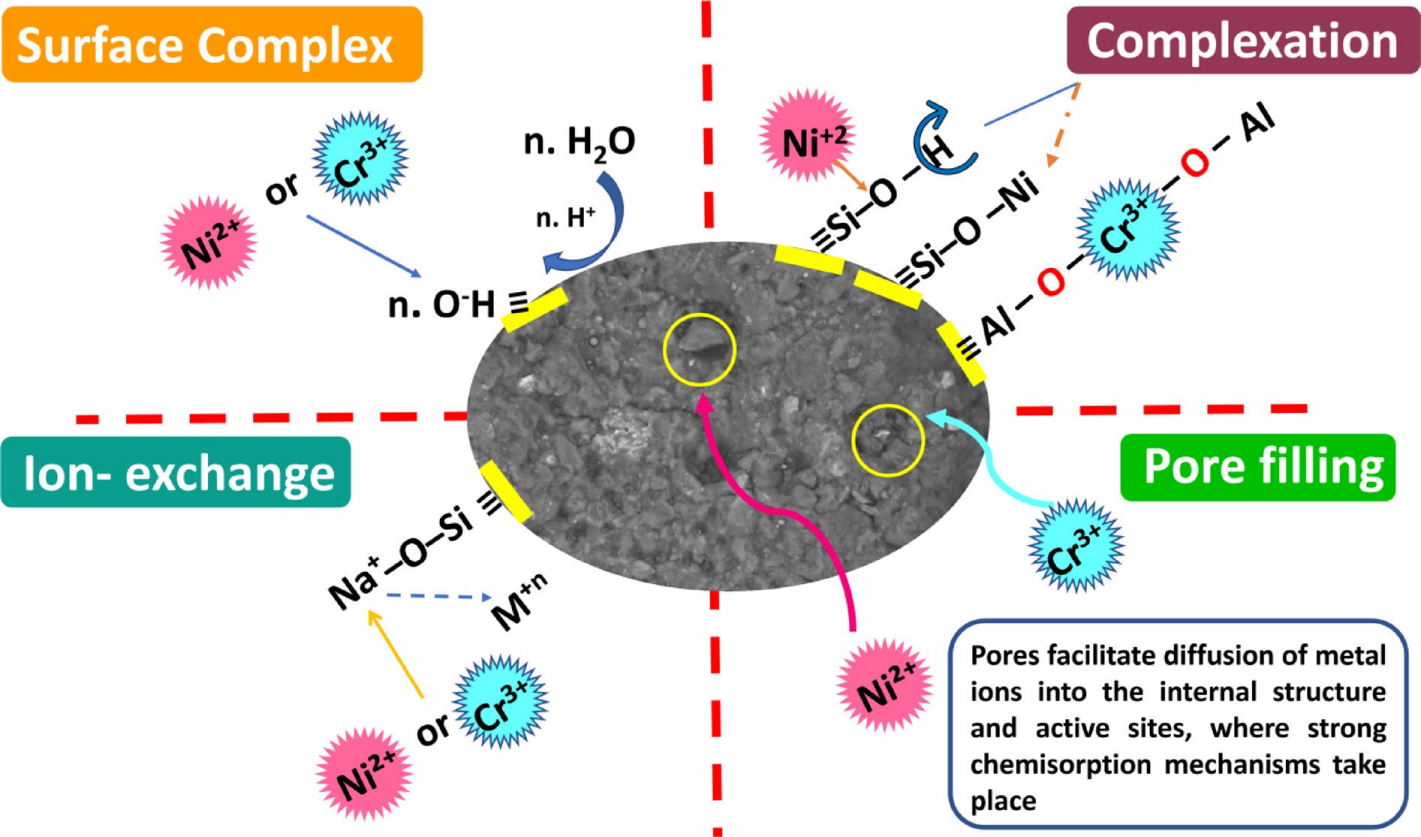
Proposed mechanism for adsorption of Ni(II) and Cr(III) by foamed and non-foamed geopolymer.

Based on the FTIR and SEM results mentioned above, the mechanism of adsorption has been suggested. The probable mechanisms of Ni(II) and Cr(III) by foamed and non-foamed geopolymer adsorbents can be seen in Fig. 21 involved different interactions in the adsorption process, including:

(i) Surface complex, (ii) Complexation (iii) Ion Exchange and (iv) Pore filling:

1. Surface Complexation: Metal ions form chemical bonds with surface functional groups (–OH, –Si–O⁻, –Al–O⁻) on the geopolymer^55^.
2. Inner-Sphere Complexation: Metal ions form strong coordination bonds with oxygen atoms in the aluminosilicate network of the geopolymer^56^.
3. Ion- Exchange : Metal cations, e.g., Ni²⁺, Cr³⁺, replace alkali ions (Na⁺, K⁺) in the geopolymer matrix^56^.
4. Pore filling: metal ions diffuse into the porous structure of the geopolymer. These pores possibly trapped Ni²⁺ and Cr³⁺ ions in mesoporous and facilitated the transport of metals into the internal structure and active sites, where strong chemisorption has occurred^57^.

The results obtained from FTIR analysis of UMFG and FMFG geoadsorbents before and after the adsorption of Ni(II) and Cr(III) revealed that O–H, Si–O–T, and Si–O or Al–O functional groups play a vital role in the adsorption mechanism. These functional groups act as active sites for metal ion binding, promoting both ion exchange and surface complexation. The surface hydroxyl group shifted from 3622 cm^−1^ before to 3620 cm^−1^ after adsorption, and also the intensity decreased compared to both geoadsorbents prior to adsorption, suggesting a coordination reaction likely occurred between Ni^+2^ or Cr^+3^ and –OH group present on the adsorbent surface, resulting in a stable metal–hydroxyl complex^51^. In addition, asymmetric stretching vibration of Si–O–T(T = Si or Al) shifted from 1002 to 1003 cm^−1^ before to 1006 cm^−1^ after adsorption, meanwhile, a new peak was formed at 1028 and 1029 cm^−1^ is presumably due to possible complexation between Ni, and Cr ions and Si–O⁻ or Al–O⁻ forming inner-sphere complexes^55^. The ion exchange mechanism involves the replacement of alkali metal ions (e.g., Na^+^ or K^+^) originally present in the geopolymer matrix with the heavy metal ions and strong chemical bonds form, making the process more irreversible.

After adsorption, the SEM micrographs indicate a remarkable change of the morphology for UMFG and FMFG. The surface morphology of both sorbents was found to be smooth, and the pores were completely filled with the metal ions after sorption, indicating that the sorbent was loaded with Ni(II) and Cr(III) ions and the metal ions were adsorbed onto functional groups located within the internal pores of the adsorbent.

### Comparison of the obtained adsorption capacity with the previously developed adsorbents in the literature

**Table 6:** Adsorption of Ni^+2^, and Cr^+3^ onto several adsorbent materials.

HMIs	Adsorbent	q_max_ (mg/g)	Isotherm Study	Kinetics Study	Thermodynamic Study	pH	Equilibrium Time	Dosage of Adsorbent	Co (ppm)	Ref.
Ni(II)	Zeolite 4 A	7.90	Langmuir	Pseudo 1 st order	------------	4.0	4 h	5 g	50–300	^58^
	Montmorillonite	10.45	Langmuir	Pseudo 2nd order	Exothermic	5.7	3 h	2 g	10–50	^59^
	Red Mud	13.69	Langmuir	Pseudo 1 st order	Endothermic	5.0	4 h	10 g	3–50	^49^
	Activated Carbon	17.24	Langmuir	Pseudo 2nd order	------------	5.0	4 h	0.2 g	10–25	^6^
	UMFG	28.34	Freundlich	Pseudo 2nd order	Endothermic	5.0	2 h	0.1 g	5–200	**This study**
	FMFG	38.46	Freundlich	Pseudo 2nd order	Endothermic	5.0	2 h	0.1 g	5–200	**This study**
Cr(III)	Bentonite	0.570	Freundlich	-----------	Exothermic	3.5	24 h	0.5 g	0.52–5.2.52.2 × 10^−8^	^60^
	Kaolin	0.898	Langmuir	Pseudo 2nd order	-----------	4.5	2 h	0.5 g	20–300	^7^
	Bagasse fly ash	4.35	Freundlich	-----------	Exothermic	5.0	40 min	10 g	5–70	^8^
	Volcanic Tuff-GP	19.3	Langmuir	Pseudo 2nd order	Endothermic	5.0	2 h	0.25 g	10–160	^31^
	UMFG	29.81	Freundlich	Pseudo 2nd order	Endothermic	5.0	3 h	0.1 g	5–200	**This study**
	FMFG	39.42	Freundlich	Pseudo 2nd order	Endothermic	5.0	3 h	0.1 g	5–200	**This study**

As can be seen in Table 6, the adsorbents utilized in this study exhibit higher adsorption capacity than the results obtained by other investigations in literature. These results indicated that the developed adsorbents demonstrated promising capabilities in removing heavy metal pollutants, making them a suitable candidate for removing other poisonous metal ions.

### Limitations of our study

The study’s primary limitation lies in its examination of metakaolin-fly ash geopolymer (both foamed and non-foamed) regarding batch experiments only, limited range of heavy metals tested, and lack of long-term regeneration performance evaluation and their effectiveness in regenerating and reusing as adsorbents for Ni(II) and Cr(III) ions from aqueous solutions. The recovery of geopolymer after heavy metal uptake necessitates additional treatment, as the physical and chemical changes during adsorption can compromise its effectiveness. Efficient regeneration strategies are thus essential; however, these can be complex and resource-intensive. Further research is required to ensure sustained performance without significant degradation of adsorption capacity while also considering the environmental impact of regeneration methods for overall sustainability. Collaboration between researchers and industry is crucial to developing cost-effective and eco-friendly regeneration techniques for geopolymer materials in heavy metal removal. Future research will concentrate on enhancing the properties of foamed and non-foamed geopolymer composites made from natural and/or industrial wastes and on developing robust regeneration techniques to promote environmentally sustainable heavy metal adsorption and remediation.

## Conclusion

The current article focused on the possibilities of using 80% metakaolin incorporated with 20% of fly ash as a precursor material in the fabrication of porous geopolymers FMFG and UMFG (non-foamed geopolymer), which have been successfully used as efficient adsorbents for the uptake of Ni(II) and Cr(III) ions from aqueous solution. According to various experimental results, the following conclusions were drawn:

- The specific surface area and pore volume of the porous geopolymer were 23.052 m^2^ g ^−1^ and 0.1454 cm^3^ g^−1^, while the non-foamed geopolymer exhibited 5.1159 m^2^ g ^−1^ and 0.0722 cm^3^ g^−1^.
- The influence of the contact time on the adsorption of Ni(II) and Cr(III) followed a similar trend for both geopolymer adsorbents, and the equilibrium time attained was within 120 and 180 min, respectively.
- The removal efficiency increases with increasing the adsorbent dosage and contact time, while it decreases as the initial Ni(II) and Cr(III) ions concentration increases.
- It was found that the porous geopolymer exhibits higher removal efficiency of Ni(II) and Cr(III) when compared to non-foamed geopolymer.
- At the optimum parameters, the maximum adsorption capacity of the FMFG was found to be 39.42 mg g^−1^ for Cr(III) and 38.46 mg/g for Ni(II) at an initial concentration of 200 mg/L as compared with the non-foamed geopolymer.
- The sorption of Cr(III) and Ni(II) onto both geoadsorbents is more consistent with pseudo-second-order kinetic models and is a fitting of the Freundlich isotherm model, confirming that the sorption mechanism is mainly chemisorption.
- The negative values of ΔG° indicated the spontaneous nature of the adsorption process, and the positive values of entropy (ΔS°) mean a random increase during adsorption. The positive enthalpy (ΔH°) values confirm that the adsorption is endothermic.
- Future work could focus on testing the adsorbents in real wastewater samples, investigating the reusability and stability over multiple cycles, the adsorption behavior for a broader range of contaminants, and the application of this technology on large scales.

**Fig. 22:**
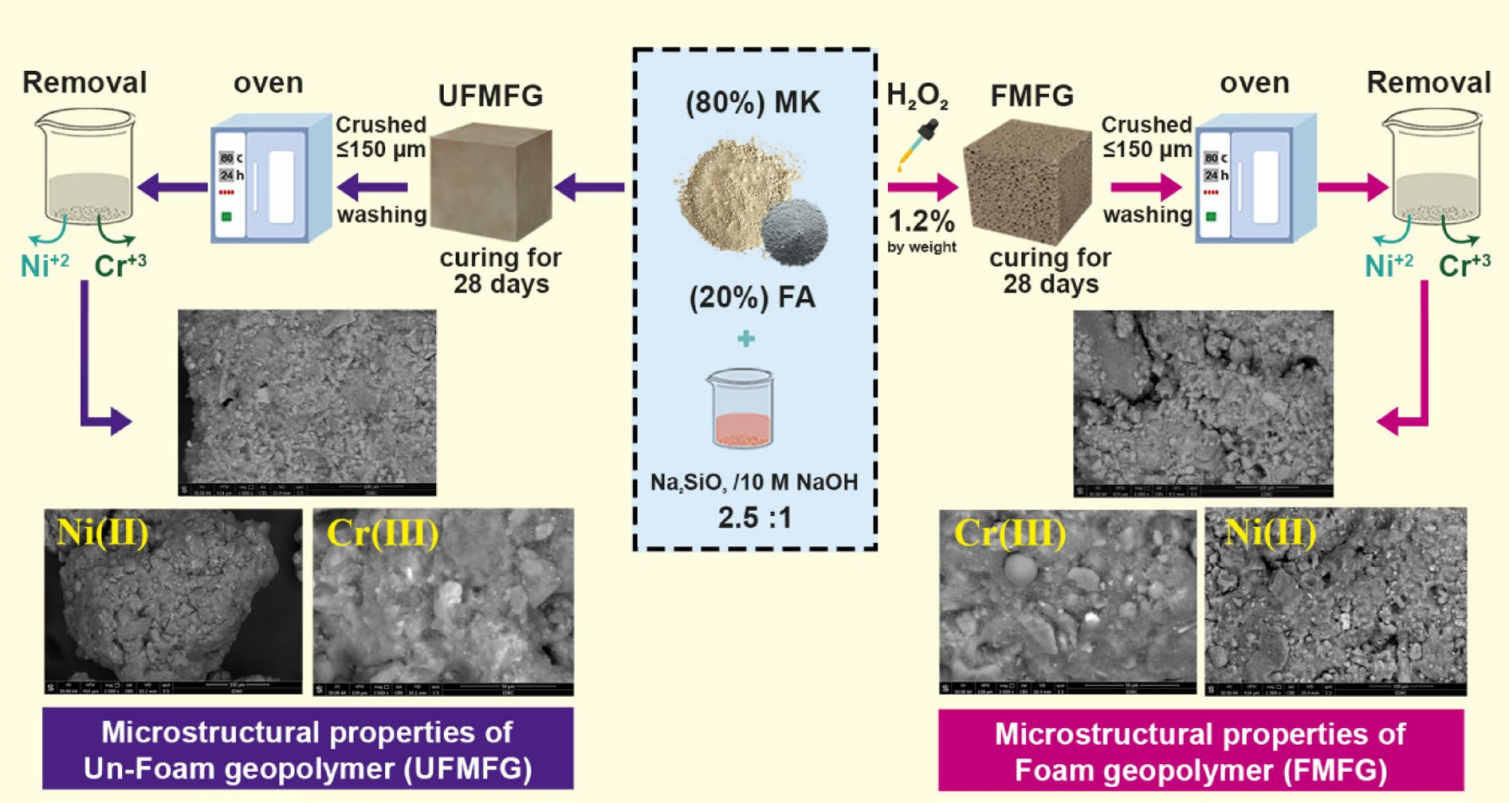
Schematic diagram of sequestration process of Ni(II) and Cr(III) ions from aqueous solution using foamed and non-foamed geopolymer based on metakaolin- fly ash.

Figure 22 schematically shows the process of sequestering Ni(II) and Cr(III) ions from aqueous solution using foamed (FMFG) and non-foamed (UMFG) geopolymer composites synthesized from 80% MK and 20% FA.

## Supplementary Information

Below is the link to the electronic supplementary material.

Supplementary Material 1

## Data Availability

The datasets used and/or analyzed during the current study are available from the corresponding author upon reasonable request.
