# Supplementary material for: Highly effective sequestration of Ni(II) and Cr(III) ions from aqueous solution using foamed and non-foamed metakaolin based geopolymer
Source: Sci Rep. 2025 Nov 10;15:39366. doi: 10.1038/s41598-025-24676-3 (PMC12603146; doi:10.1038/s41598-025-24676-3)
Supplement: Supplementary file 1 — Supplementary Material 1 [file 41598_2025_24676_MOESM1_ESM.docx]

**Highlights**

- Efficient removal of Ni (II) and Cr (III) ions from aqueous media using foam and non-foam metakaolin/fly ash-based geopolymers
- The effect of pH, contact time, dosage, and initial concentration was appraised to control the [adsorption capacity](https://www.sciencedirect.com/topics/engineering/adsorption-capacity); the equilibrium time attained was within 120 and 180 minutes for Ni (II) and Cr (III), respectively.
- The high adsorption capacity of 38.46 mg/g for Ni^+2^ and 39.42 mg/g for Cr^+3^ ions by FMFG was achieved.
- After adsorption, the IR spectra showed a new absorption band at 1028 and 1029 cm⁻¹, indicating Ni(II) and Cr(III) loading onto the adsorbent. Meanwhile SEM analysis were significantly altered, with smoother surfaces and white spots after metal uptake, suggesting the entrapment of Ni (II) and Cr(III) onto the sorbent after adsorption.
- Adsorption mechanisms relies on the chemisorption and are involved complexation of sorption.
